# S100A9 is indispensable for survival of pneumococcal pneumonia in mice

**DOI:** 10.1371/journal.ppat.1011493

**Published:** 2023-07-19

**Authors:** Lena Ostermann, Benjamin Seeliger, Sascha David, Carolin Flasche, Regina Maus, Marieke S. Reinboth, Martin Christmann, Konstantin Neumann, Korbinian Brand, Stephan Seltmann, Frank Bühling, James C. Paton, Johannes Roth, Thomas Vogl, Dorothee Viemann, Tobias Welte, Ulrich A. Maus

**Affiliations:** 1 Division of Experimental Pneumology, Hannover Medical School, Hannover, Germany; 2 Clinic for Pneumology, Hannover Medical School, Hannover, Germany; 3 German Center for Lung Research, Hannover, Germany; 4 Institute of Intensive Care Medicine, University Hospital Zurich, Zurich, Switzerland; 5 Institute of Clinical Chemistry, Hannover Medical School, Hannover, Germany; 6 Labopart Medical Laboratories, Dresden and Chemnitz, Germany; 7 Research Centre for Infectious Diseases, Department of Molecular and Biomedical Science, University of Adelaide, Adelaide, Australia; 8 Institute of Immunology, University of Münster, Münster, Germany; 9 Department of Pediatric Pneumology, Allergology and Neonatology, Hannover Medical School, Hannover, Germany; 10 Translational Pediatrics, Department of Pediatrics, University Hospital Würzburg, Germany; 11 Cluster of Excellence RESIST (EXC 2155), Hannover Medical School, Hannover, Germany; 12 Center for Infection Research, University Würzburg, Germany; Trinity College Dublin, IRELAND

## Abstract

S100A8/A9 has important immunomodulatory roles in antibacterial defense, but its relevance in focal pneumonia caused by *Streptococcus pneumoniae* (*S*. *pneumoniae*) is understudied. We show that S100A9 was significantly increased in BAL fluids of patients with bacterial but not viral pneumonia and correlated with procalcitonin and sequential organ failure assessment scores. Mice deficient in S100A9 exhibited drastically elevated Zn^2+^ levels in lungs, which led to bacterial outgrowth and significantly reduced survival. In addition, reduced survival of S100A9 KO mice was characterized by excessive release of neutrophil elastase, which resulted in degradation of opsonophagocytically important collectins surfactant proteins A and D. All of these features were attenuated in *S*. *pneumoniae*-challenged chimeric WT→S100A9 KO mice. Similarly, therapy of *S*. *pneumoniae*-infected S100A9 KO mice with a mutant S100A8/A9 protein showing increased half-life significantly decreased lung bacterial loads and lung injury. Collectively, S100A9 controls central antibacterial immune mechanisms of the lung with essential relevance to survival of pneumococcal pneumonia. Moreover, S100A9 appears to be a promising biomarker to distinguish patients with bacterial from those with viral pneumonia.

**Trial registration**: Clinical Trials register (DRKS00000620).

## Author summary

Acute bacterial infections of the lower respiratory tract remain a major cause of morbidity and mortality worldwide. They are usually associated with massive recruitment of neutrophilic granulocytes to the lungs, which release specific proteins called S100A8 and S100A9 in addition to many other inflammatory mediators. S100A8 and S100A9 belong to the family of calcium-binding proteins and preferentially form S100A8/A9 heterodimers. When released by neutrophils upon infection, S100A8/A9 proteins are capable of binding several metal ions. In this report, we show that S100A9 is significantly elevated in the lungs of patients with bacterial but not viral pneumonia, suggesting that this alarmin might be a promising new biomarker in bacterial pneumonia. In addition, we show in a preclinical mouse model that S100A8/A9 reduces bacterial growth *in vivo* and *in vitro* by binding important metal ions like manganese and zinc. The observed drop in bacterial loads was further accompanied by reduced release of neutrophil elastase in lungs of mice, which in turn protected the lungs of mice from infection-driven lung damage. Collectively, S100A8/A9 proteins are important antibacterial effector proteins of the lung and thus indispensable for survival of pneumococcal pneumonia.

## Introduction

*Streptococcus pneumoniae* (*S*. *pneumoniae*) is the most prevalent pathogen causing community-acquired pneumonia (CAP) in humans [1–4]. Despite available vaccines, CAP caused by *S*. *pneumoniae* still represents a life-threatening clinical condition especially in young children (< 5 years) and elderly or immunocompromised individuals [5–9]. Particularly, resident alveolar macrophages and neutrophils play important roles in the elimination of invading pneumococci by releasing reactive oxygen species, neutral serine proteases and other cytosolic proteins including myeloperoxidase (reviewed in [10–13]).

S100A8 and S100A9 are part of the EF-hand superfamily of Ca^2+^ binding proteins comprising approximately 25 members [14–16]. The Ca-binding EF-hand motif was first described in 1973 by Kretsinger et al. and consists of two alpha-helices connected by a loop which is able to bind Ca^2+^ ions [17]. Both S100 proteins are of low molecular weight (8 kDa and 14 kDa) and account for ~40% of the cytosolic protein content of neutrophilic granulocytes [13,15,18]. Several important immunomodulatory properties have been linked to heterodimeric S100A8/S100A9 which is collectively termed calprotectin (CP). Given that CP serves as a chelating agent for essential divalent metal ions, it directly affects microbial and fungal growth and the activity of matrix metalloproteinases [19–23]. S100A8/A9 has also been shown to interact with target proteins such as Toll-like receptor (TLR) 4 or receptor for advanced glycation end products (RAGE) in order to increase host inflammatory activities including inflammatory cell migration and expression of pro-inflammatory cytokines, as well as regulation of the NLRP3 inflammasome [24–31]. In this context, previous reports suggested that lack of S100A8/A9 impaired CD11b dependent neutrophil recruitment to the site of infection [32–35].

The role of S100A8 and S100A9 in bacterial infections caused by *S*. *pneumoniae* is still understudied. Here we characterized the role of functional S100A8/A9 deficiency in a model of pneumococcal pneumonia in mice. We found that the heterodimer regulates essential antibacterial immune mechanisms by controlling levels of divalent cations in the lung and thereby inhibiting bacterial outgrowth. Second, neutrophil elastase (NE) release by recruited neutrophils was limited in the presence but not the absence of S100A9, with major impact on NE-dependent degradation of surfactant proteins SP-A and SP-D and overall survival. Finally, S100A9 appears to be a promising biomarker to distinguish bacterial from viral pneumonia patients.

## Results

### S100A9 is significantly increased in lungs of patients with bacterial but not viral acute respiratory distress syndrome

**Fig 1:**
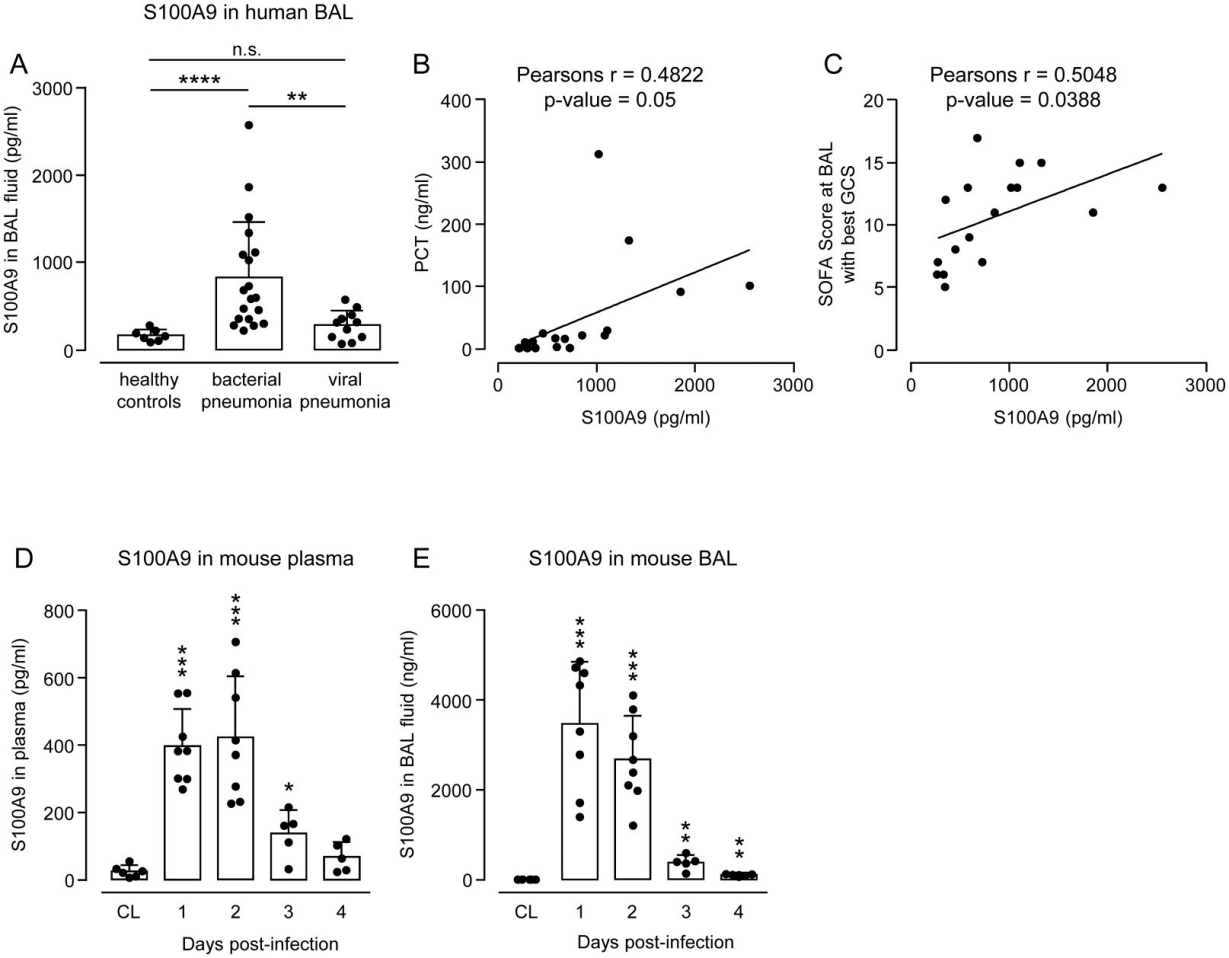
Endogenous S100A9 levels and its correlation with clinical parameters in BALF of pneumonia patients, as well as S100A9 levels in plasma and BALF of *S*. *pneumoniae*-infected WT mice. (A) S100A9 levels were determined in BALF of patients with bacterial or viral pneumonia and healthy controls. Data are shown as mean ± SD of n = 7–19 patients. Significant correlation between BAL fluid levels of S100A9 and procalcitonin (B) or SOFA scores at BAL with best GCS (C) in patients with bacterial pneumonia. GCS, Glasgow coma score (n = 17 patients). WT mice were left untreated (CL) or were infected with *S*. *pneumoniae*. Endogenous S100A9 levels were determined in plasma (D) and BAL fluids (E) of mice at days 1, 2, 3 and 4 post-infection by ELISA. Data are shown as mean ± SD of n = 5–8 mice per time point and treatment group and are representative of two independently performed experiments. *p ≤ 0.05; **p ≤ 0.01; ***p ≤ 0.001, ****p ≤ 0.0001 compared to controls or patients with viral pneumonia (Mann-Whitney U test).

S100A8/A9 heterodimer, also known as calprotectin (CP), is a protein complex serving as a biomarker for different inflammatory diseases like inflammatory bowel disease (i.e. Crohn’s disease and ulcerative colitis), and rheumatoid arthritis [36–39]. Acute bacterial infections are characterized by massive recruitment of neutrophilic granulocytes into the lung interstitium and alveolar air spaces. S100A9 and its binding partner S100A8 are known to be primarily released by neutrophils. Therefore, we wondered whether one of these small molecules would allow discrimination of bacterial from viral pneumonia/acute respiratory distress syndrome (ARDS). Interestingly, we found significantly increased S100A9 protein levels in bronchoalveolar lavage fluid (BALF) of patients with bacterial but not viral pneumonia (see Table 1 for patient details), relative to healthy controls (Fig 1A).

**Table 1:** Patient characteristics and pathogenic agents in the bronchoalveolar lavage fluid of patients with severe bacterial or viral ARDS.

Patient characteristics	Bacterial ARDS	Viral ARDS
Total patients	19	11
Male	12	8
Female	7	3
Age mean (range)	52.3 (22–75)	53.9 (20–87)
Non-smokers (ex or never)	10	5
Current smokers	9	6
Spectrum of pathogens		
Influenza		9
Human metapneumovirus		1
Herpes simplex virus		1
Gram-positive (*S*. *pneumoniae*, Group A *Streptococcus*, *S*. *aureus*)	10	
Gram-negative (*Legionella sp*., *Klebsiella sp*., *E*.*coli*)	6	
unclassified	3	

Moreover, S100A9 protein levels were positively correlated with C reactive protein (CRP) in BALF of patients with bacterial but not viral pneumonia (Pearson’s r = 0.54, p-value = 0.03 for bacterial versus viral pneumonia with Pearson’s r = -0.76, p-value = 0.01) (S1A and S1B Fig). In addition, we found a positive correlation between S100A9 protein and procalcitonin (PCT, Pearson’s r = 0.48 p-value = 0.05) (Fig 1B) as well as sequential organ failure assessment (SOFA) Score at BAL with best Glasgow Coma Scale (GCS, Pearson’s r = 0.50, p-value = 0.04) (Fig 1C) in patients with bacterial pneumonia. These data support the view that S100A9 may serve as biomarker to distinguish bacterial from viral pneumonia/ARDS.

### S100A9 levels increase in plasma and BAL fluid of WT mice after challenge with *S*. *pneumoniae*

We then determined levels of S100A9 protein in WT mice challenged with *S*. *pneumoniae*. At baseline, we found low levels of S100A9 protein in plasma and BALF of WT mice, which increased significantly in response to pneumococcal challenge with peak values observed at days 1–2 post-infection (Fig 1D and 1E).

### Lack of S100A9 aggravates pneumococcal pneumonia in mice

**Fig 2:**
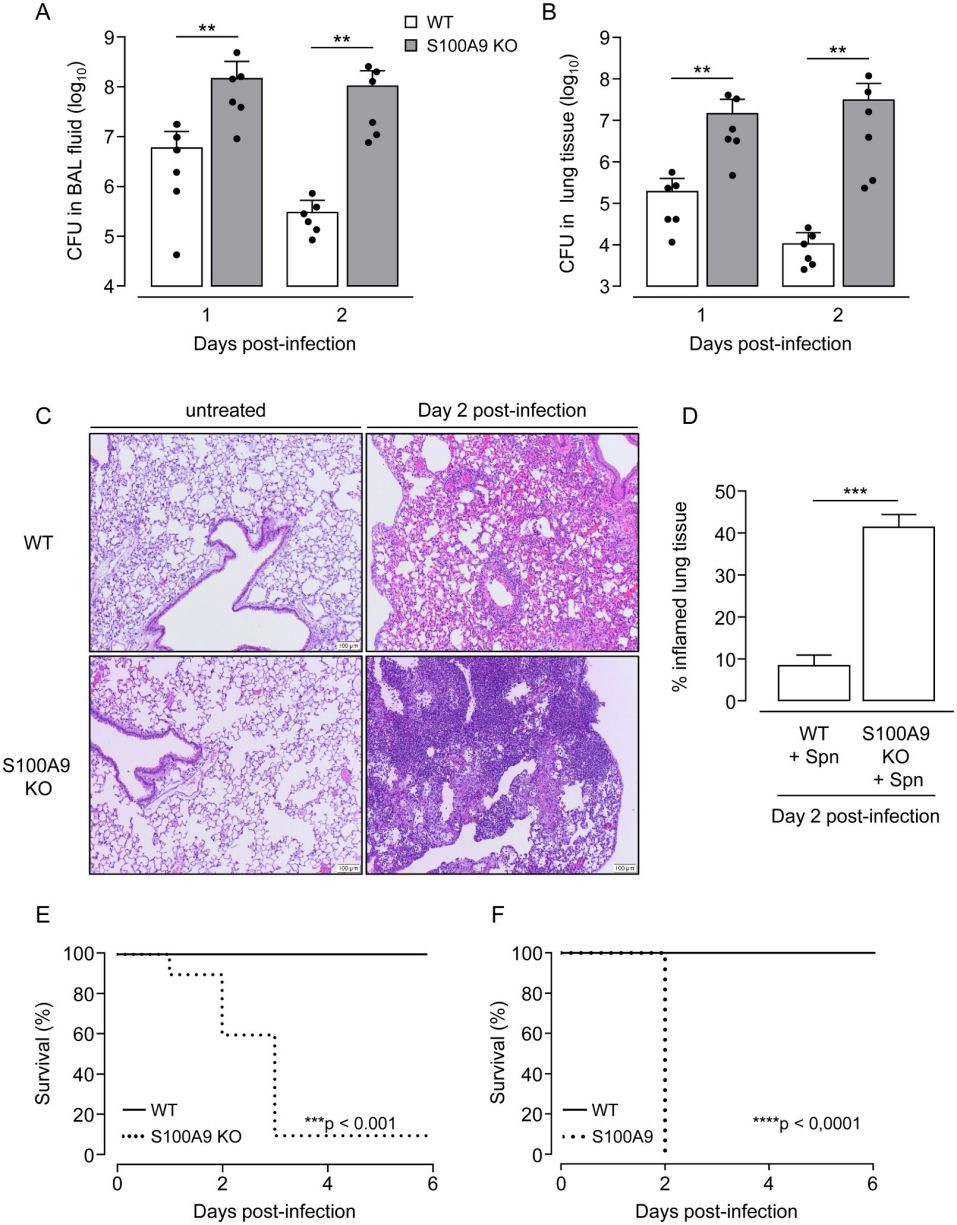
Effect of S100A9 deficiency on outcome in pneumococcal pneumonia. WT mice (white bars) and S100A9 KO mice (grey bars) were infected orotracheally with *S*. *pneumoniae* and were analyzed at indicated time points. (A,B) Bacterial loads in BALF (A) and lung tissue (B) at 24 hours and 48 hours post-infection. Values are shown as mean ± SD (n = 6 mice per time point and treatment group) and are representative of two independent experiments. (C) Lung histopathology of untreated (left panels, scale bar: 100 μm) and *S*. *pneumoniae*-infected (right panels, scale bar: 100 μm) WT and S100A9 KO mice on day 2 post-infection. Illustrations (C) are representative of n = 4 mice per group. (D) Percent inflamed lung tissue of *S*. *pneumoniae*-challenged WT and S100A9 KO mice at day 2 post-infection (n = 3 mice per treatment group). (E,F) Survival analysis of WT and S100A9 KO mice challenged with a low (6 x 10^6^ CFU) (E) or increased (10^7^ CFU) infection dose of *S*. *pneumoniae* (n = 9 mice per group). **p ≤ 0.01; ***p ≤ 0.001, ****p ≤ 0.0001 compared to WT mice (Mann-Whitney U test, t-Test, log-rank test).

To further characterize the role of S100A9 in lung antibacterial immunity, we made use of S100A9 knockout mice. These mice are also deficient of S100A8 protein, most likely due to decreased S100A8 protein stability in the absence of S100A9 [25,32,40]. Infection of S100A9 KO mice with *S*. *pneumoniae* led to significantly increased bacterial loads in BALF and lung tissue on days 1 and 2 post-infection when compared to *S*. *pneumoniae*-infected WT mice (Fig 2A and 2B). Histopathological examination of hematoxylin/eosin (HE) stained lung tissue sections revealed a normal lung architecture in untreated WT and S100A9 KO mice (Fig 2C, left panels). Following *S*. *pneumoniae* infection, lungs of S100A9 KO mice developed severe lobar bronchopneumonia characterized by consolidated infiltrates of interstitial and alveolar neutrophils and interstitial and alveolar edema, while lungs of *S*. *pneumoniae*-infected WT mice showed much less severe signs of bronchopneumonia (Fig 2C and 2D). Corresponding to the severe histopathology, S100A9 KO mice challenged with a low infection dose of 6 x 10^6^ CFU *S*. *pneumoniae* showed a significantly decreased survival of just 10% by day 3 post-infection, compared to 100% survival for *S*. *pneumoniae*-challenged WT mice (Fig 2E). Challenge of WT and S100A9 KO mice with a higher infection dose (10^7^ CFU *S*. *pneumoniae*) confirmed the striking susceptibility of S100A9 KO mice to pneumococcal challenge (Fig 2F).

### CD11b expression is upregulated on recruited neutrophils of *S*. *pneumoniae*-infected WT and S100A9 KO mice

**Fig 3:**
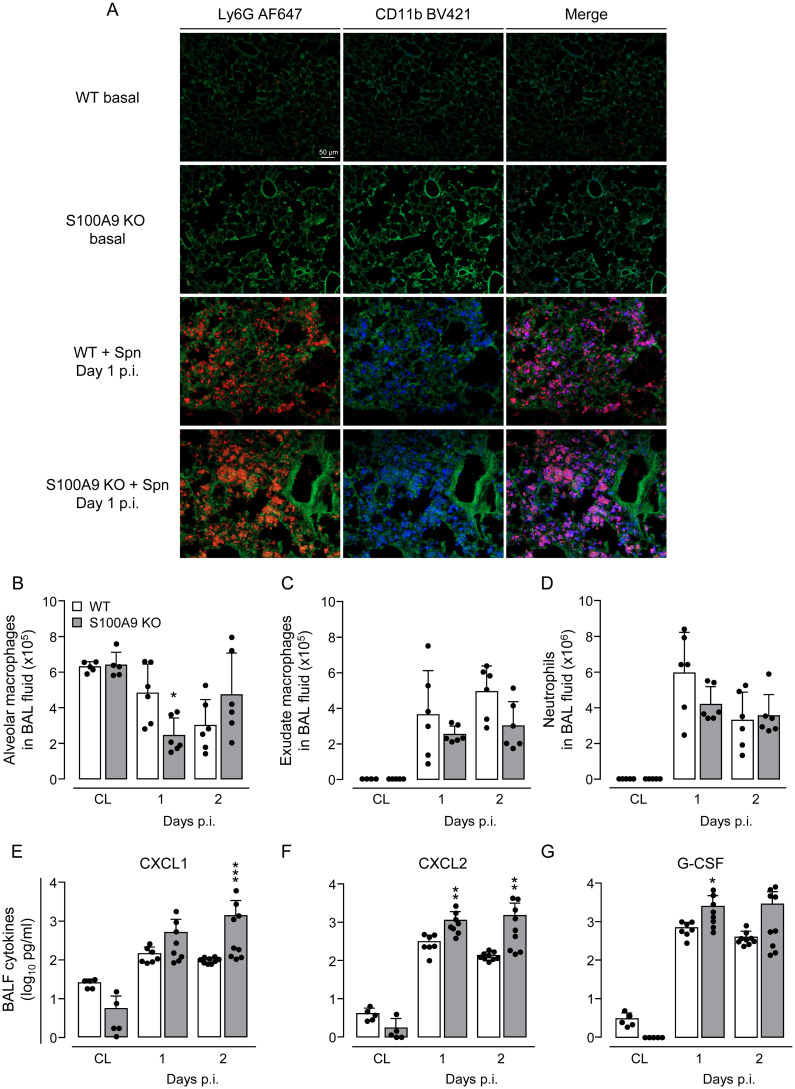
Leukocyte recruitment and cytokine expression in lungs of *S*. *pneumoniae*-challenged WT and S100A9 KO mice. (A) Representative immunofluorescence analysis of recruited neutrophils and corresponding CD11b expression in frozen lung tissue sections of untreated and *S*. *pneumoniae*-infected WT and S100A9 KO mice on day 1 post-infection. Lung tissue sections were stained with AF647-conjugated anti-Ly6G antibody (red) and BV421-conjugated anti-CD11b antibody (blue). Green autofluorescence was recorded for topographical purposes. Merge profiles identify Ly6G and CD11b-positive neutrophils in lung tissue (purple fluorescence staining) (n = 4 mice per group, scale bar: 50 μm). (B-D) Differential leukocyte counts in BAL fluid of WT and S100A9 KO mice challenged with *S*. *pneumoniae* on day 1 and 2 post-infection (n = 5–6 mice per time point and treatment group). (E-G) Cytokine expression in BALF of untreated and *S*. *pneumoniae*-infected WT and S100A9 KO mice (n = 5–9 mice per time point and treatment group). Values are shown as means ± SD and are representative of two independent experiments. *p ≤ 0.05; **p ≤ 0.01; ***p ≤ 0.001 compared to WT mice (Mann-Whitney U test).

Previous studies in S100A9 KO mice suggested compromised neutrophil immigration in *S*. *pneumoniae*-infected lungs due to decreased CD11b upregulation on neutrophils following bacterial challenge [32,33,35]. Therefore, we examined lung neutrophil recruitment by immunofluorescence analysis of CD11b and Ly6G (a specific neutrophil marker) on frozen lung tissue sections of untreated and *S*. *pneumoniae*-infected S100A9 KO and WT mice (Fig 3A). Under baseline conditions, virtually no Ly6G^+^ and CD11b^+^ neutrophils could be detected in the lungs of mice of either experimental group. However, on day 1 post-infection, multiple Ly6G/CD11b double-positive neutrophils were detected in the lung interstitium and alveolar air space of both WT and S100A9 KO mice (Fig 3A) with no apparent difference between groups. These findings illustrate that S100A9 KO neutrophils are fully capable of expressing the β2 integrin CD11b and are equally well recruited into the bronchoalveolar space in pneumococcal pneumonia compared to WT mice. Analysis of differential leukocyte counts in BALF of *S*. *pneumoniae*-challenged WT and S100A9 KO mice revealed similar numbers of recruited neutrophils and exudate macrophages in both experimental groups (Fig 3C and 3D). These findings were corroborated by FACS analyses of leukocyte subsets in BALF and lungs of untreated and *S*. *pneumoniae*-challenged WT and S100A9 KO mice with CFU counts dropping in WT but not S100A9 KO mice (S2 Fig). As expected, our FACS analyses confirmed Ly6G and CD11b expression on recruited neutrophils as also determined by immunofluorescence staining of neutrophils (Figs 3A, S2B and S2G). Numbers of alveolar macrophages were only transiently reduced in S100A9 KO mice on day 1 post-infection, while reaching normal levels on day 2 post-infection (Fig 3B). Luminex-based analyses of pro- and anti-inflammatory cytokines showed significantly increased levels of CXCL1, CXCL2 and G-CSF in BALF of S100A9 KO mice as compared to WT mice on days 1 and 2 after pneumococcal infection (Fig 3E–3G). Moreover, opposed to WT mice, S100A9 KO mice showed substantially increased BAL fluid levels of pro-inflammatory TNF-α and IL-1β and anti-inflammatory IL-10 on day 2 post-infection (S3A–S3C Fig).

### Effect of S100A9 deletion on bacterial phagocytosis and burst induction in professional phagocytes in vitro

Based on the finding that defective antibacterial responses in S100A9 KO mice were not due to defective lung neutrophil recruitment, we next questioned whether phagocytosis as well as burst induction might be impaired in professional phagocytes of S100A9 KO mice as compared to WT mice. As shown in supplemental S4A and S4B Fig, we did not observe any significant difference in bacterial phagocytosis of *S*. *pneumoniae* by alveolar macrophages or bone marrow-derived neutrophils between experimental groups. Also, neutrophils purified from S100A9 KO and WT mice responded with a similar respiratory burst induction to *S*. *pneumoniae*-infection *in vitro* (supplemental S4C Fig).

### Defective lung antibacterial immunity is restored in S100A9 KO mice after reconstitution with WT bone marrow

**Fig 4:**
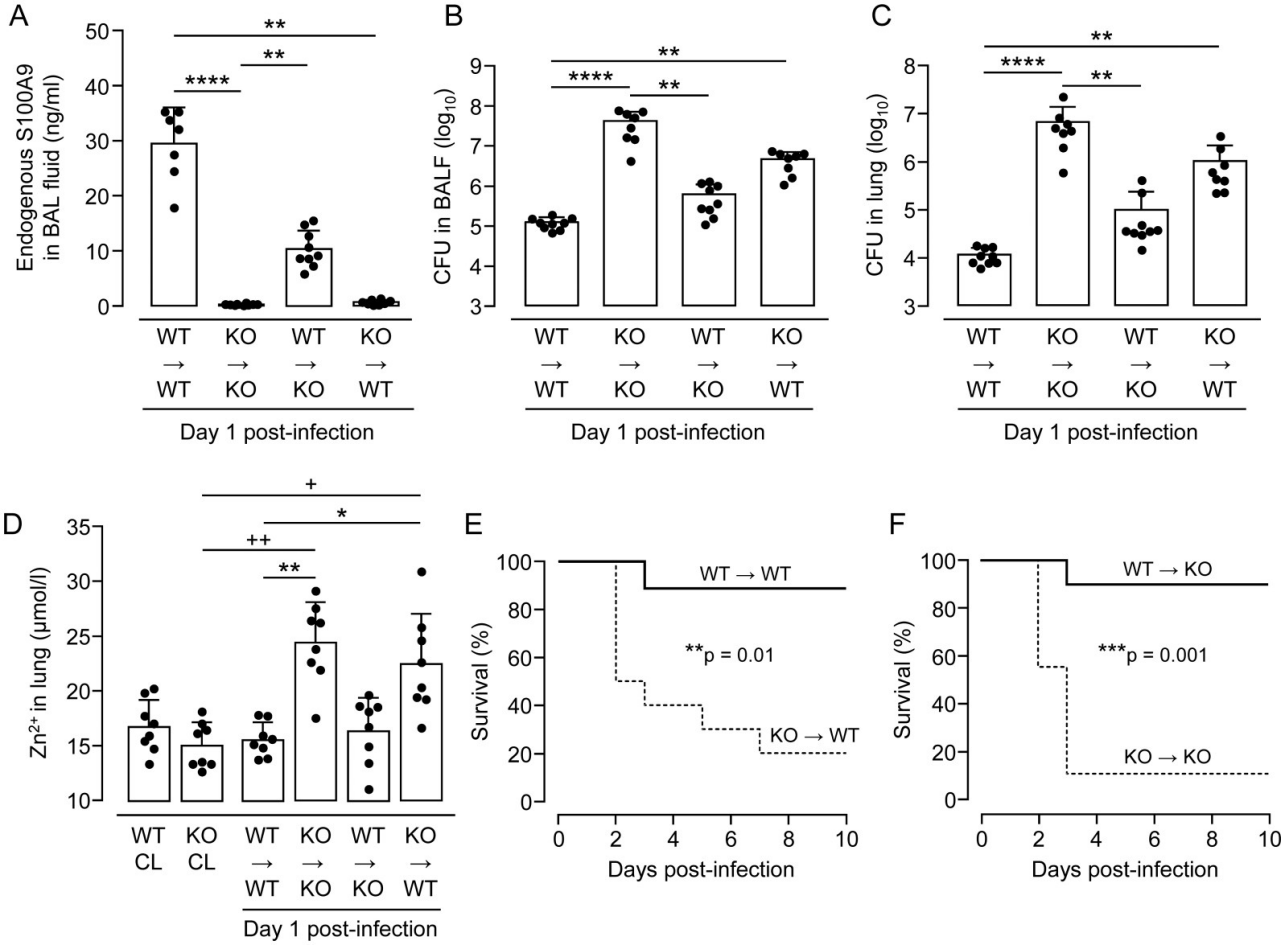
Effect of S100A9 deletion or reconstitution on lung antibacterial immunity in *S*. *pneumoniae*-infected bone-marrow chimeric mice. (A) Endogenous S100A9 levels in BALF of chimeric mice on day 1 after pneumococcal challenge (n = 7–10 mice per group). (B,C) Bacterial loads in BAL fluids (B) and lung tissue (C) of *S*. *pneumoniae*-challenged chimeric mice on day 1 post-infection (n = 8–9 mice per group). (D) Zinc levels in lungs of *S*. *pneumoniae*-infected chimeric mice on day 1 post-infection, relative to controls (n = 8 mice per treatment group). Data are shown as means ± SD and are representative of two independently performed experiments. (E,F) Survival of chimeric mice infected with *S*. *pneumoniae* during an observation period of 10 days (n = 9–10 mice per group). The data represent two independent experiments. *p ≤ 0.05; **p ≤ 0.01; ***p ≤ 0.001, ****p ≤ 0.0001 compared to chimeric controls, +p ≤ 0.05; ++p ≤ 0.01 compared to untreated controls (Kruskal-Wallis test, log-rank test).

We found that S100A9 is of major importance for lung antibacterial immunity against *S*. *pneumoniae* in mice. Since S100A9 is primarily released by activated myeloid cells such as neutrophils [13,18], we questioned whether reconstitution of S100A9 KO mice with bone marrow from WT mice would restore the defective lung antibacterial immunity in KO mice. Therefore, we established four groups of bone marrow chimeric mice, i.e. WT→WT, WT→KO, KO→WT, and KO→KO chimeric mice in order to evaluate under which experimental conditions lung antibacterial immunity would be restored to WT conditions. As shown in Fig 4A, S100A9 protein levels were drastically elevated in BALF of WT→WT chimeras following *S*. *pneumoniae*-infection, whereas S100A9 protein was not detectable in BALF of KO→KO chimeric mice. As expected, WT→KO chimeric mice showed significantly increased S100A9 levels in BALF post-infection, while KO→WT chimeras again demonstrated low levels of S100A9 protein in BALF (Fig 4A). These studies illustrate that S100A9 protein is derived from hematopoietic cells immigrating the lung, and its detection within the bronchoalveolar space coincided with alveolar immigrating neutrophils. At the same time, we found low CFU counts in BALF and lung tissue of WT→WT mice (day 1 post-infection), while KO→KO mice showed drastically increased bacterial loads in BALF and lung tissue (Fig 4B and 4C). Of note, WT→KO chimeric mice exhibited significantly decreased CFU in BALF and lung tissue, whereas KO→WT chimeric mice again exhibited significantly increased bacterial CFU in BALF and lung tissue when compared to WT→WT controls (Fig 4B and 4C). These data collectively illustrate that S100A9 is derived from the hematopoietic system and critically regulates lung antibacterial immunity in mice.

### Effect of S100A9 deficiency on Zn^2+^ levels in lungs of *S*. *pneumoniae*-infected mice

S100A8/A9 has been shown previously to play an important role as a chelating protein complex, binding various divalent metal ions with consequences for bacterial replication [19–21]. However, its role in regulating metal ion concentrations in pneumococcal pneumonia is poorly defined. Therefore, we analyzed Zn^2+^ levels in lungs of untreated and *S*. *pneumoniae*-infected WT and S100A9 KO mice, as well as the various groups of chimeric mice. At baseline, WT and S100A9 KO mice showed similar Zn^2+^ levels in lung homogenates (~15 μmolar), and pneumococcal challenge did not alter Zn^2+^ levels in lungs of WT→WT mice (Fig 4D). However, Zn^2+^ levels increased significantly in lungs of *S*. *pneumoniae*-challenged KO→KO mice (Fig 4D), but dropped significantly in lungs of *S*. *pneumoniae*-challenged chimeric WT→KO mice, as opposed to KO→WT chimeras, which again demonstrated significantly increased Zn^2+^ levels in their lungs (Fig 4D). Consequently, 90% of WT→WT mice survived pneumococcal pneumonia, whereas *S*. *pneumoniae*-infected KO→WT chimeras showed approximately 20% survival by day 7 post-infection. Similarly, WT bone marrow reconstitution in S100A9 KO mice rescued mice from fatal pneumonia (survival at 90%), while > 80% KO→KO mice succumbed to pneumococcal pneumonia (Fig 4E and 4F). These data show that S100A9 critically contributes to survival of pneumococcal pneumonia in mice.

### Treatment of *S*. *pneumoniae*-infected S100A9 KO mice with mutant S100A8/A9 protein substantially improves lung antibacterial immunity

**Fig 5:**
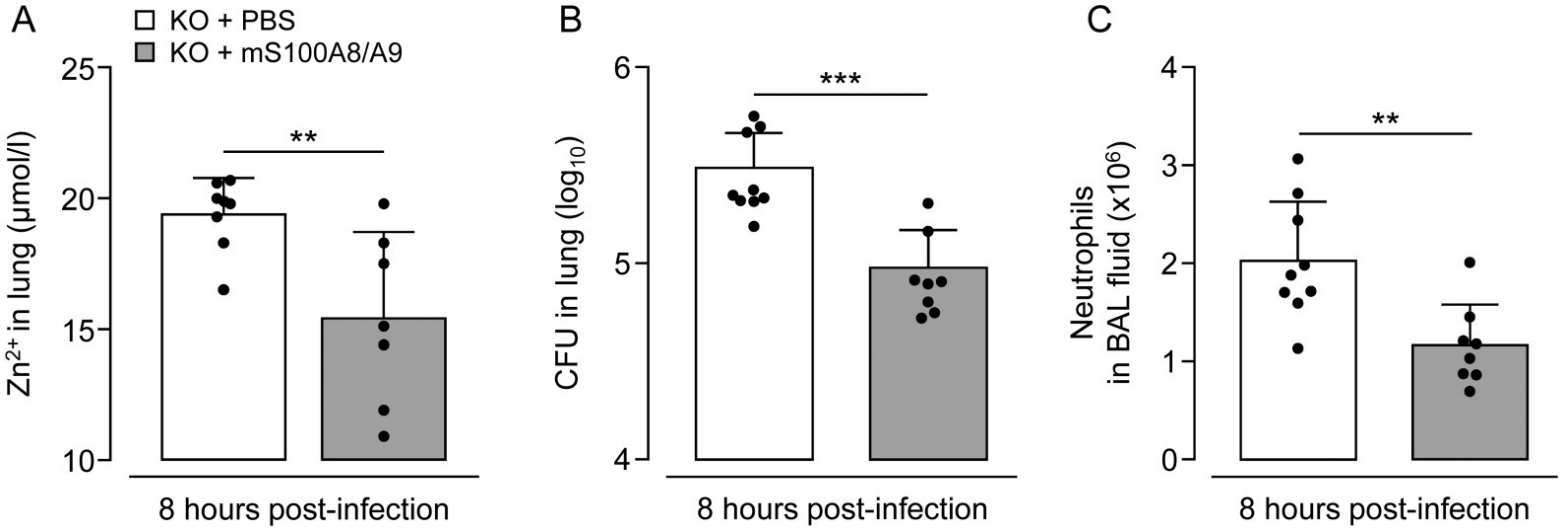
Effect of mutant S100A8/A9 protein application on antibacterial responses and Zn^2+^ levels in lungs of *S*. *pneumoniae*-infected S100A9 KO mice. S100A9 KO mice were orotracheally infected with *S*. *pneumoniae* and therapeutically treated with mutant S100A8/A9 protein or vehicle i.p., as indicated. (A) Zn^2+^ levels in lungs of *S*. *pneumoniae*-infected S100A9 KO mice treated with mutant S100A8/A9 protein or vehicle i.p. at 8 hours post-infection. (B) Bacterial loads in lung at 8 hours after pneumococcal challenge. (C) Numbers of recruited neutrophils in BAL fluids of mutant S100A8/A9 or vehicle treated *S*. *pneumoniae*-infected S100A9 KO mice at 8 hours post-infection. Values are shown as mean ± SD of n = 7–9 mice per time point and treatment group and are representative of two independent experiments. **p ≤ 0.01; ***p ≤ 0.001 compared to vehicle treated mice (Mann-Whitney U test).

In selected experiments, we aimed to explore the efficacy of S100A8/A9 protein to improve lung antibacterial immunity in S100A9 KO mice after challenge with *S*. *pneumoniae*. We employed a mutant S100A9 protein carrying amino acid exchanges at distinct sites within the calcium-binding EF-hand II of the protein, thereby creating a mutant murine heterodimeric S100A8/A9 protein with increased biological half-life [41–43]. As shown in Fig 5A, Zn^2+^ levels did not increase in response to pneumococcal challenge of S100A9 KO mice in the presence of mutant S100A8/A9, while a substantial increase in Zn^2+^ levels was observed in vehicle-treated, *S*. *pneumoniae*-infected S100A9 KO mice (Fig 5A). At the same time, S100A9 KO mice treated with mutant S100A8/A9 protein demonstrated significantly improved lung bacterial clearance at 8 hours post-challenge, opposed to vehicle treated S100A9 KO mice (Fig 5B). Consistent with these results, we found substantially reduced numbers of recruited neutrophils in BAL fluids of mutant S100A8/A9 treated, *S*. *pneumoniae*-infected S100A9 KO mice (Fig 5C). These data demonstrate that application of S100A8/A9 effectively restores lung antibacterial immunity against *S*. *pneumoniae* in a highly susceptible host.

### S100A8/A9 inhibits growth of *S*. *pneumoniae in vitro*

**Fig 6:**
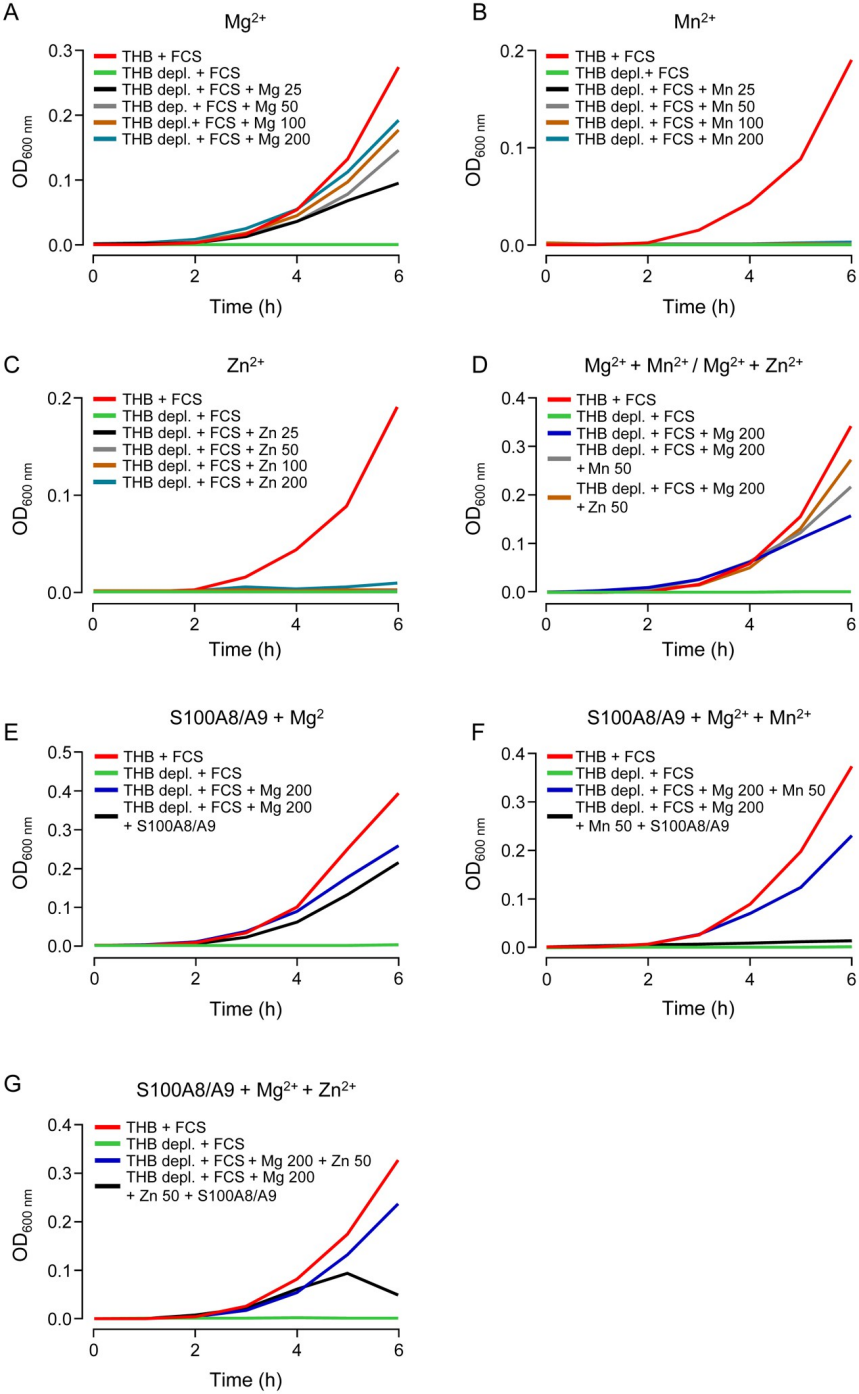
Growth of *S*. *pneumoniae* under defined growth conditions *in vitro*. To achieve defined growth conditions, THB medium was treated with Chelex 100 resin. (A-G) Pneumococci were not able to grow in depleted THB medium + 1% FCS (A-G, green line). (A-C) Depleted THB medium was supplemented with increasing concentrations of certain divalent cations. (A) Addition of MgCl_2_ to depleted THB medium + 1% FCS restored growth of *S*. *pneumoniae* in a dose dependent manner. Treatment of depleted THB + 1% FCS with different concentrations of either MnSO_4_ (B) or ZnCl_2_ (C) alone was not able to retrieve pneumococcal growth *in vitro*. (D) Pneumococcal growth in medium supplemented with 200 μM Mg^2+^ + 50 μM Mn^2+^ or 200 μM Mg^2+^ + 50 μM Zn^2+^ was nearly identical to pneumococcal growth in normal THB + 1% FCS. Additionally, depleted THB + 1% FCS medium supplemented with either Mg^2+^ alone (E), Mg^2+^ + Mn^2+^ (F) or Mg^2+^ + Zn^2+^ (G) was further supplemented with recombinant S100A8/A9 (50 μg/ml). *S*. *pneumoniae* was then added and bacterial growth was monitored hourly over a time period of six hours. Untreated THB medium supplemented with 1% FCS served as positive control in all growth experiments (A-G). The Data are representative of two independently performed experiments.

To prove that the metal ion chelating properties of S100A8/A9 contributed to inhibition of pneumococcal growth, we next established a Chelex-based growth medium in order to deplete defined essential transition metals for bacterial growth studies *in vitro*. As shown in Fig 6, in the absence of divalent metal ions, *S*. *pneumoniae* was not able to grow even in the presence of 1% FCS (green line in Fig 6A–6G). In a next step, our Chelex-treated (i.e., divalent cation depleted) medium was supplemented with defined divalent metal ions to identify those facilitating pneumococcal growth *in vitro*. As shown in Fig 6A, addition of MgCl_2_ restored pneumococcal growth in a dose-dependent manner, whereas the addition of MnSO_4_ and ZnCl_2_ alone was not able to restore pneumococcal growth in Chelex-treated medium supplemented with 1% FCS (Fig 6B and 6C).

Based on the finding that Mg^2+^ was essential for pneumococcal growth *in vitro*, we next established defined growth conditions to evaluate the additional effect of Mn^2+^ or Zn^2+^ on pneumococcal growth *in vitro* using Chelex-treated medium supplemented with magnesium + 1% FCS. As outlined in Fig 6D, pneumococcal growth in medium supplemented with Mn^2+^ (50 μM, grey line in Fig 6D) or Zn^2+^ (50 μM, each in the presence of 200 μM Mg^2+^ + 1% FCS) (orange line in Fig 6D) restored pneumococcal growth to a similar extent as observed in the control THB + 1% FCS growth medium (red line, Fig 6A–6G). Addition of recombinant S100A8/A9 (50 μg/ml) to Chelex-depleted medium supplemented with Mg^2+^ alone had only a very minor effect on pneumococcal growth (black line, Fig 6E). Importantly however, when recombinant S100A8/A9 was added to Chelex-treated growth medium supplemented with Mn^2+^ or Zn^2+^ (each at 50 μM in the presence of 200 μM Mg^2+^ + 1% FCS), pneumococcal growth was significantly inhibited *in vitro* (black lines, Fig 6F and 6G). These data show that S100A8/A9 primarily inhibits pneumococcal growth by chelation of divalent cations Mn^2+^ and Zn^2+^, rather than Mg^2+^.

### Neutrophil elastase dependent degradation of surfactant proteins A and D in lungs of S100A9 deficient mice

**Fig 7:**
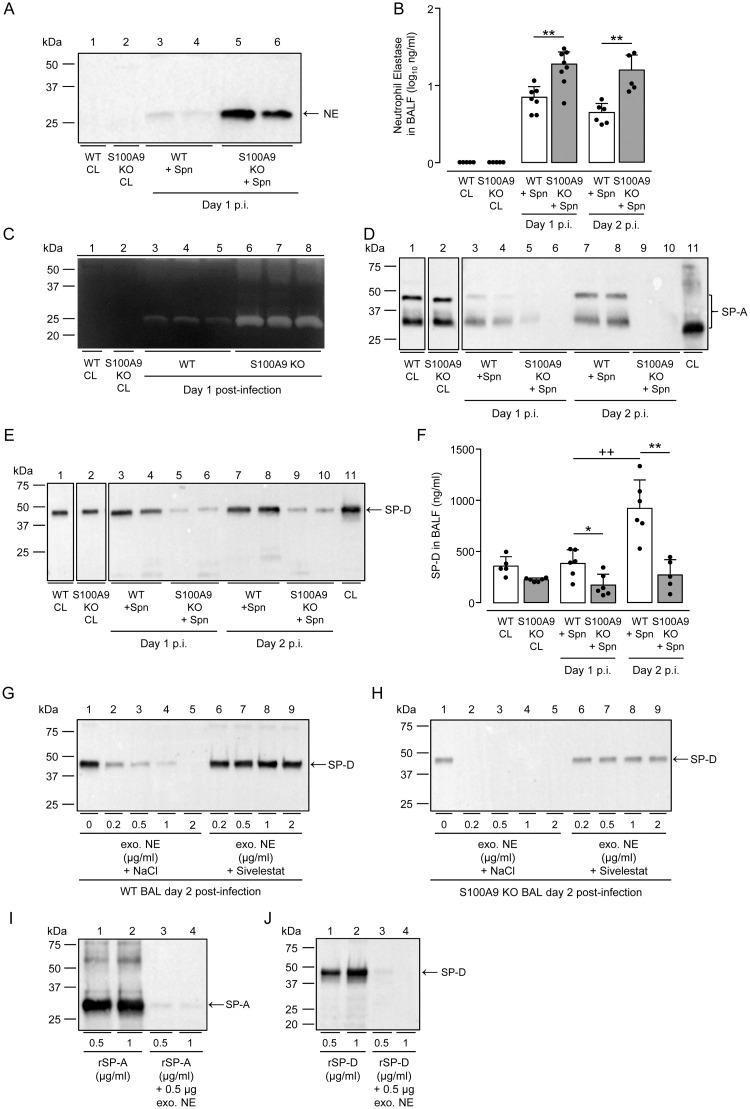
NE dependent degradation of alveolar collectins SP-A and SP-D in the lungs of *S*. *pneumoniae*-challenged S100A9 KO mice. (A) Neutrophil elastase in BAL fluids of untreated or *S*. *pneumoniae*-infected WT versus S100A9 KO mice at day 1 post-infection as indicated. Equal amounts of BAL protein (15 μg) were used for western blot analysis. (B) Quantification of NE protein levels in BAL fluids of mice of the respective treatment groups by ELISA. (C) Caseinolytic activity in BALFs of untreated and *S*. *pneumoniae*-infected WT and S100A9 KO mice on day 1 post-infection. (D,E) SP-A (D) and SP-D (E) protein levels in BALFs of untreated and *S*. *pneumoniae*-challenged WT versus S100A9 KO mice on day 1 and day 2 post-infection, as indicated. Recombinant SP-A or SP-D protein serving as positive control (lane 11 in D,E). (F) Quantification of SP-D protein in BAL fluids of the respective treatment groups. Data are shown as mean ± SD of 5–8 mice per time point and group and are representative of two independently performed experiments. (G,H) Incubation of exogenous NE with BALF of *S*. *pneumoniae*-infected WT or S100A9 KO mice led to degradation of SP-D (G, H, lanes 1–5), while pre-incubation of NE with specific inhibitor Sivelestat prevented SP-D degradation *in vitro* (G,H, lanes 6–9). (I,J) Just 0.5 μg NE are sufficient to degrade rSP-A (I) and rSP-D (J) *in vitro*. *p ≤ 0.05; **p ≤ 0.01 compared to WT mice, ++p ≤ 0.01 compared to day 2 (Mann-Whitney U test).

We recently found that *S*. *pneumoniae* infection of alpha-1 antitrypsin (AAT) deficient mice triggered substantial degradation of alveolar collectins surfactant protein A (SP-A) and D (SP-D) in a neutrophil elastase dependent manner, with fatal consequences for the infected host [44]. These studies implied that alveolar depletion of collectins might be a principal consequence of neutrophil-dominated lung infections such as pneumococcal pneumonia. Since S100A9 KO mice also succumbed to neutrophil-dominated severe pneumonia, we next examined release of neutrophil elastase in BALF supernatant of *S*. *pneumoniae*-infected WT as compared to S100A9 KO mice. As shown in Fig 7A ,NE was not detectable by western blotting in BALF of untreated WT and S100A9 KO mice (lanes 1, 2). Upon *S*. *pneumoniae* infection, NE was only weakly detectable in BALF of WT mice, but strongly upregulated in BALF of S100A9 KO mice (Fig 7A, lanes 3–6). Subsequent ELISA analyses confirmed strong differences in BAL fluid NE levels between WT and S100A9 KO mice (Fig 7B). Moreover, casein-based zymography confirmed the proteolytic activity of NE in BALF of S100A9 KO mice as opposed to WT mice (Fig 7C, lanes 3–8). Importantly, increased NE activities were associated with substantial loss of SP-A (both monomeric 35 kDa and oligomeric 48 kDa protein bands) and SP–D protein contents in BALF of *S*. *pneumoniae*-challenged S100A9 KO mice, which was corroborated by ELISA (Fig 7D–7F).

To further confirm that indeed NE was responsible for the observed degradation of collectins in BALF of *S*. *pneumoniae*-challenged S100A9 KO mice, BALF of infected WT and S100A9 KO mice (containing collectins SP-A and–D) were incubated with exogenous NE, followed by analysis of SP-D by western blotting. As shown in Fig 7G and 7H, we found that exogenous NE degraded SP-D in BALF of *S*. *pneumoniae*-infected WT and S100A9 KO mice. While SP-D protein was degraded in BAL fluids of *S*. *pneumoniae*-infected WT mice in a dose dependent manner (Fig 7G, lanes 1–5), just 0.2 μg of exogenous NE were sufficient to degrade the residual SP-D protein detected in BALF of infected S100A9 KO mice (Fig 7H, lanes 1–5). Importantly, NE-specific proteolytic activity was blocked by pre-incubating exogenous NE with the NE-specific inhibitor Sivelestat (Fig 7G and 7H, lanes 6–9). Moreover, just 0.5 μg NE was sufficient to completely degrade recombinant SP-A and SP-D (1 μg/sample) *in vitro* (Fig 7I and 7J). These data strongly support the view that neutrophil-derived NE is responsible for degradation of collectins SP-A and SP-D in BAL fluids of *S*. *pneumoniae*-infected WT and S100A9 KO mice.

### Analysis of NE and SP-A/SP-D in bone marrow chimeric WT and S100A9 KO mice

**Fig 8:**
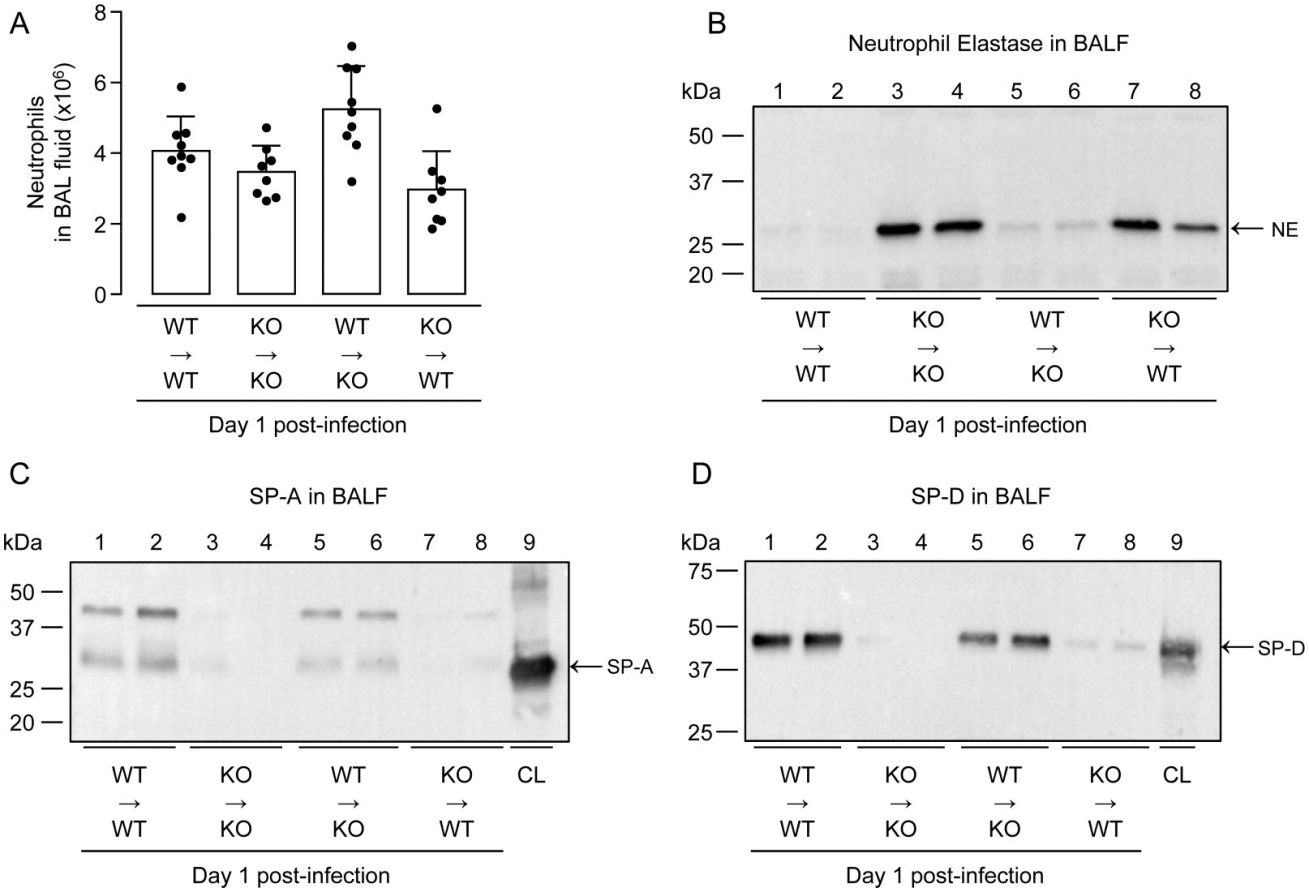
Neutrophil elastase-dependent degradation of SP-A and SP-D in lungs of bone-marrow chimeric mice. (A) Numbers of neutrophils in lungs of *S*. *pneumoniae*-challenged bone-marrow chimeric mice on day 1 post-infection. Data are shown as mean ± SD of 8–9 mice per treatment group. (B-D) Neutrophil elastase (B), SP-A (C) and SP-D (D) proteins in BAL fluids of bone-marrow chimeric WT→WT, KO→KO, WT→KO and KO→WT mice on day 1 after pneumococcal challenge as indicated. The data are representative of two independently performed experiments.

Lack of S100A9 protein was accompanied by exaggerated NE contents in BALF of *S*. *pneumoniae*-challenged KO mice, suggesting that NE release by alveolar recruited neutrophils is high in the absence of S100A9. To confirm this inverse relationship between NE and S100A9, we again made use of our chimeric mice generated by reciprocal bone marrow transplantation (BMT) of WT or S100A9 KO bone marrow into WT or KO mice. As shown in Fig 8A, numbers of BALF neutrophils were not significantly different between the four groups of chimeric mice. However, analysis of NE protein contents in BALF of chimeric mice revealed major differences between WT→WT mice with low NE contents and KO→KO mice with drastically increased NE contents, and further between WT→KO mice with low NE contents and KO→WT mice with again drastically increased NE contents, as shown in Fig 8B. These data clearly confirm that BALF NE levels are low once S100A9 sufficient neutrophils are recruited in response to *S*. *pneumoniae*, and drastically increase once S100A9 deficient neutrophils are recruited upon *S*. *pneumoniae* infection. At the same time, low levels of BALF NE were accompanied by easily detectable levels of SP-A and SP-D in WT→WT and WT→KO mice, whereas BAL fluids of KO→KO and KO→WT mice with increased NE levels completely lacked collectins SP-A and–D (Fig 8C and 8D).

### Release of neutrophil elastase depends on the presence of the pneumococcal virulence factor pneumolysin

**Fig 9:**
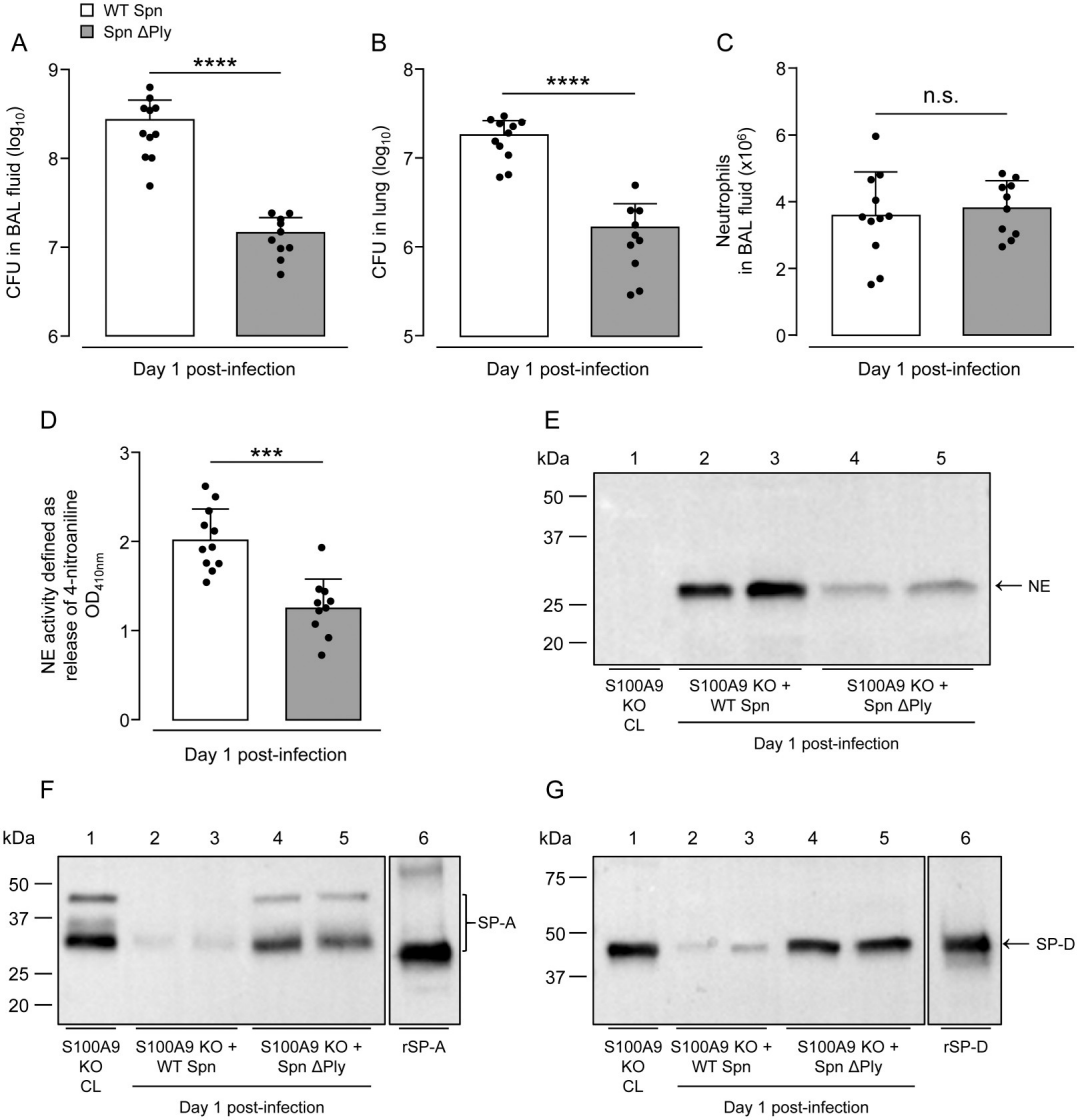
Neutrophil activation and subsequent release of NE depends on pneumococcal virulence factor pneumolysin. S100A9 KO mice were infected with WT *S*. *pneumoniae* and *S*. *pneumoniae* ΔPly and analyzed on day 1 post-infection. (A,B) Bacterial loads in BALF (A) and lung tissue (B) of the respective treatment groups on day 1 post-infection. (C) Numbers of neutrophils in lungs of S100A9 KO mice infected with WT *S*. *pneumoniae* or *S*. *pneumoniae* ΔPly on day 1 post-infection. (D) Neutrophil elastase activity of equal numbers of neutrophils recovered by bronchoalveolar lavage from mice of the respective treatment groups. Data are shown as mean ± SD of 10–11 mice per time point and group. (E-G) Western blot analysis of NE (E), SP-A (F) and SP-D (G) in BALF of WT *S*. *pneumoniae* and *S*. *pneumoniae* ΔPly-infected S100A9 KO mice on day 1 post-infection as indicated. The data are representative of two independently performed experiments. ***p ≤ 0.001, ****p ≤ 0.0001 compared to WT *S*. *pneumoniae* infected S100A9 KO mice (Mann-Whitney *U* test).

Pneumolysin (Ply) is an important cholesterol-binding virulence factor of pneumococcus, that also serves as a pathogen-associated molecular pattern (PAMP) binding to and activating professional phagocytes via TLR4 [45]. Pneumolysin may contribute to neutrophil cell death and subsequent release of neutrophil elastase [46,47]. Therefore, we questioned what effect Ply would have on the observed NE release in *S*. *pneumoniae*-challenged S100A9 KO mice. To this end, we compared bacterial CFU and NE release and activity in BAL fluids of S100A9 KO mice challenged with either Ply-sufficient or -deficient *S*. *pneumoniae*. As shown in Fig 9, S100A9 KO mice infected with *S*. *pneumoniae* ΔPly showed significantly decreased bacterial loads in BALF and lung tissue on day 1 post-infection relative to KO mice challenged with WT S. *pneumoniae*, with similar BAL neutrophil counts in both groups (Fig 9A–9C). At the same time, NE activity was substantially reduced in lysates of equal numbers of BAL neutrophils from *S*. *pneumoniae* ΔPly as compared to WT *S*. *pneumoniae*-infected S100A9 KO mice (Fig 9D). As expected, western blot analysis revealed high levels of NE in WT *S*. *pneumoniae*-infected S100A9 KO mice, but weak NE protein bands in BALF of *S*. *pneumoniae* ΔPly-infected S100A9 KO mice, with again no significant differences in BAL neutrophil counts between groups (Fig 9C and 9E). At the same time, substantial degradation of SP-A and SP-D occurred in BALF of *S*. *pneumoniae*-challenged KO mice, while both collectins were abundantly detected in BALF of *S*. *pneumoniae* ΔPly-infected S100A9 KO mice (Fig 9F and 9G). The data show that *S*. *pneumoniae*-driven neutrophil activation and subsequent degranulation of NE is dependent to a large extent on Ply and independent of S100A9.

## Discussion

**Fig 10:**
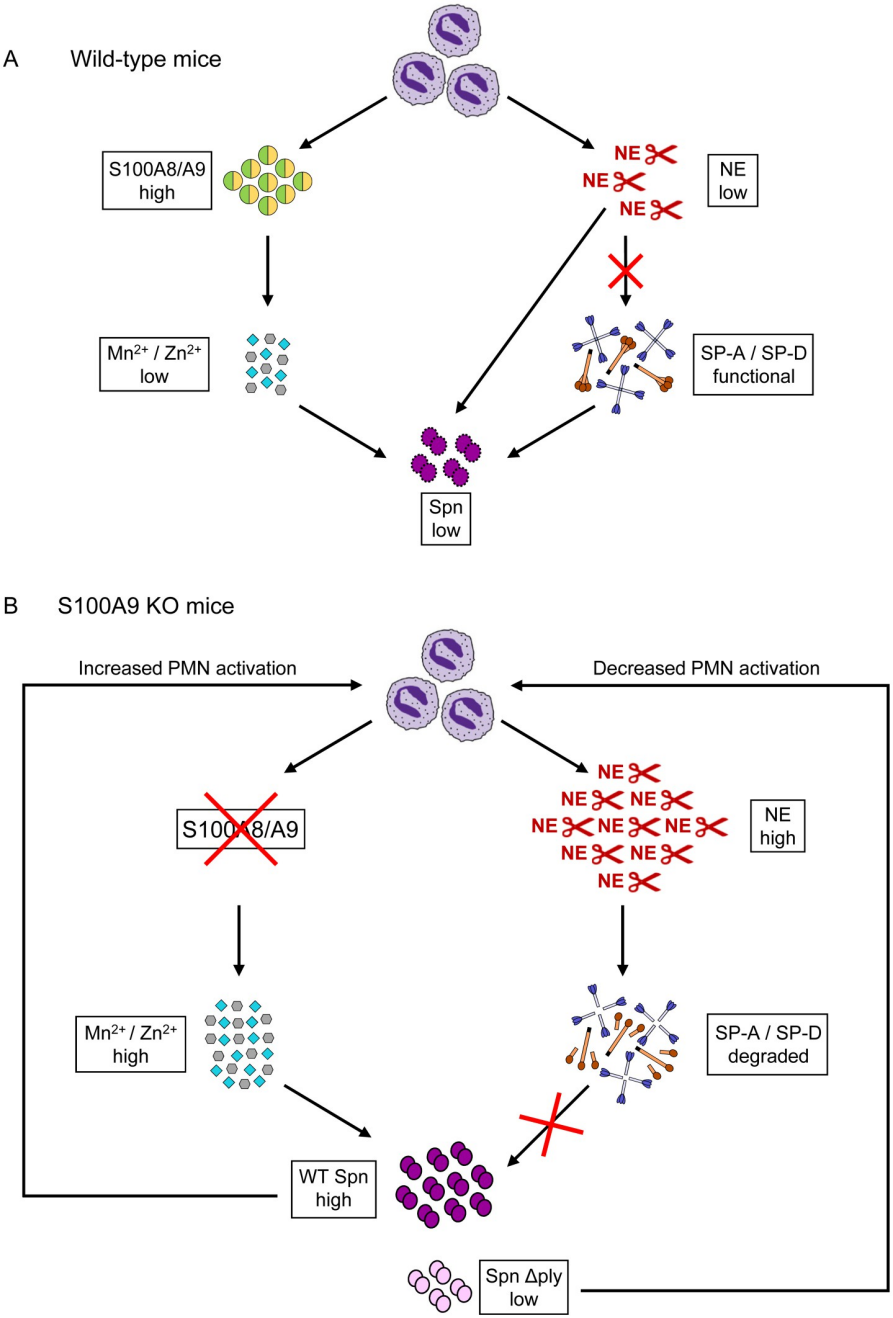
Schematic concept of how lung antibacterial immunity is affected by S100A8/A9 deficiency. (A) S100A8/A9 is released by neutrophils upon activation. By complexing divalent metal ions S100A8/A9 inhibits bacterial outgrowth. As a consequence, less neutrophil recruitment and neutrophil-dependent NE release is observed. This protects alveolar collectins SP-A and SP-D from NE-dependent degradation. (B) S100A8/A9 deficient neutrophils are recruited to the site of infection. In the absence of S100A8/A9, divalent metal ions foster bacterial outgrowth, resulting in excessive neutrophil recruitment and release of NE. As a consequence, the increased proteolytic NE burden in lungs promotes degradation of opsonophagocytically important collectins SP-A and SP-D, thereby impairing lung antibacterial immunity against *S*. *pneumoniae*. *S*. *pneumoniae*-driven neutrophil activation and NE release are diminished in infection experiments involving *S*. *pneumoniae* ΔPly.

The current study was conducted to further characterize S100A9 in lung antibacterial immunity in a well-defined model of pneumococcal pneumonia in mice. The data support an important role for S100A9 in lung innate immunity against *S*. *pneumoniae*: 1) Increased levels of S100A9 protein were found in BALF of patients with bacterial, but not viral ARDS, which were correlated with CRP, PCT and SOFA scores, suggesting S100A9 as novel biomarker for bacterial pneumonia. 2) Increased levels of S100A9 correlated with low levels of Zn^2+^ and bacterial loads in lungs of *S*. *pneumoniae*-infected mice, and S100A8/A9 heterodimer inhibited Zn^2+^ and Mn^2+^ dependent pneumococcal growth *in vitro*. 3) Lack of S100A9 (and thus heterodimeric S100A8/A9) led to increased NE dependent degradation of surfactant proteins SP-A and -D, which was accompanied by poor outcome in KO mice, and corrected in chimeric S100A9 KO mice harboring the hematopoietic system of WT mice. Together, S100A8/A9 limits pneumococcal outgrowth in focal pneumonia by chelation of divalent cations, which in turn limits pneumolysin-driven release of neutrophil-derived NE and subsequent NE-dependent degradation of surfactant proteins, thus favoring survival of pneumococcal pneumonia (Fig 10).

The heterodimeric S100A8/A9 protein (Calprotectin, CP) has been assigned several immunomodulatory activities and also serves as biomarker in inflammatory diseases including, but not limited to, ulcerative colitis, Crohn’s disease and rheumatoid arthritis [36–39,48,49]. Since biomarkers are urgently needed for a better differentiation of pathogen-induced ARDS [50], we wondered whether CP may be a suitable biomarker for bacterial pneumonia. Previous studies found that serum CP levels allowed discrimination between bacterial and viral pneumonia patients with even greater accuracy than procalcitonin and heparin binding protein (HBP) [51]. On the other hand, a most recent study reported major proteolytic and oxidative degradation of calprotectin in BALF of patients with cystic fibrosis (CF). Moreover, NE and MPO activities correlated with CP-derived peptides, again supporting a rapid disassembly of CP *in vivo* [52]. Bacterial infections of the lung are typically neutrophil-dominated lung diseases and characterized by increased proteolytic activities (including NE), as shown in this study and most recently by our group [44]. Therefore, it is conceivable that an increased proteolytic activity developing subsequent to alveolar neutrophil immigration may favor degradation of CP, thus rendering it unsuitable as biomarker for bacterial pneumonia. Notably, an additional caveat for the use of CP itself as biomarker for bacterial infections is the finding that high concentrations of CP may also be detected in plasma of patients with severe viral infections such as COVID 19 due to high amounts of immature neutrophils released from the bone marrow [53].

Therefore, we wondered whether not CP, but rather S100A9 protein itself would be an appropriate biomarker for discrimination between bacterial and viral ARDS. Others have reported increased S100A9 levels in the serum of patients with CAP, which positively correlated with inflammatory cytokines and blood routine parameters [54]. Moreover, we previously found increased S100A9 mRNA levels in professional phagocytes in an ARDS-like mouse model, and S100A9 was significantly upregulated in BALF of ARDS patients [55–58]. Capitalizing on these findings, we show that levels of S100A9 were significantly increased in BALF of patients with bacterial (including pneumococcal) but not viral pneumonia and positively correlated with CRP, PCT and SOFA scores. As two recently published guidelines clearly made a recommendation against PCT as primary diagnostic biomarker protein [59,60], these data further support the potential importance of S100A9 as novel biomarker for bacterial pneumonia.

The role of S100A8/A9 in experimental models of bacterial lung infection is controversial. Detrimental effects of S100A8/A9 deficiency were observed in *S*. *aureus* and *K*. *pneumoniae* pneumonia, and increased bacterial loads and inflammatory responses were observed in S100A9 KO mice in a model of invasive pneumococcal disease (IPD) [61–63]. In contrast, others reported prolonged survival and reduced bacterial loads in the absence of S100A8/A9 protein in a model of type 3 (strain 6303) IPD, while suggesting that elevated zinc levels in host tissue led to zinc-dependent toxicity against *S*. *pneumoniae* [64]. However, use of an invasive pneumococcal strain (serotype 3) which is known to rapidly disseminate from the lung to the circulation implies that lung-directed antibacterial effector mechanisms such as alveolar neutrophil-derived S100A9 are difficult to address in such a model. Therefore, in the current study we have employed a non-invasive pneumococcal strain (serotype 19) causing focal pneumonia without sepsis [65]. For a similar reason, bacteria were directly applied into the lower airways of mice in the current study, while intranasal delivery of pneumococci as reported previously [64] may more rapidly cause bacterial dissemination and thus invasive pneumococcal disease. However, to directly compare the results of both studies, experiments employing both invasive and non-invasive pneumococcal strains would need to be performed in parallel. Moreover, other factors like the origin or sex of mice may also contribute to the susceptibility of mice to pneumococcal challenge.

Previous studies found that neutrophil recruitment was diminished in the absence of S100A8/A9 in preclinical models of invasive pneumococcal disease [34,63] and tuberculosis [33], presumably due to a lowered CD11b expression on elicited neutrophils [33,34,66]. Inhibition of S100A8 and S100A9 by neutralizing antibodies also attenuated lung neutrophilia in a model of serotype 3 IPD, surprisingly without affecting bacterial clearance or survival of mice [34]. Impaired neutrophil recruitment was also observed in S100A9 KO mice in a model of IPD due to significantly reduced G-CSF levels [63]. However, in the current study, no significant differences between WT and S100A9 KO mice were found in numbers of recruited neutrophils or expression of β2 integrin CD11b on elicited neutrophils in our model of focal pneumonia, as judged by immunofluorescence or FACS analysis. These findings are further corroborated by our extensive analyses of bone-marrow chimeric mice demonstrating similar numbers of recruited neutrophils in either of the four different experimental groups. Collectively, our study supports the view that S100A9 is not critical for lung neutrophil recruitment during pneumococcal pneumonia.

It is known that S100A8/A9 is able to prevent bacterial and fungal growth by binding essential divalent metal ions like Zn^2+^ and Mn^2+^ [19–22,62]. Here we show that S100A8/A9 plays an important role in lowering concentrations of divalent metal ions in defined pneumococcal growth experiments *in vitro*, and that deficiency of S100A9 (and thus of heterodimeric S100A8/A9) was accompanied by increased levels of divalent metal ions in pneumococcal pneumonia, leading to bacterial outgrowth. In contrast, previous studies suggested that S100A8/A9 may contribute to worsening of IPD by complexing and thereby reducing zinc-mediated toxicity against the pathogen, hence fostering bacterial outgrowth [64]. Opposed to this, other groups showed that S100A8/A9 effectively inhibited *Staphylococcus aureus* outgrowth in locally confined tissue abscesses, due to complexing of essential Mn^2+^ and Zn^2+^ ions [20,21]. In the current study, we were able to show that systemic application of exogenous S100A8/A9 protein significantly improved lung antibacterial immunity against *S*. *pneumoniae* in highly susceptible S100A9 KO mice by lowering Zn^2+^ levels in lungs of mice accompanied by attenuated bacterial outgrowth. Again, the contradictory results regarding the role of S100A8/A9 in lowering divalent metal ions may be related to the different pathogenicity profiles of the pneumococcal strains and thereof developing infection phenotypes employed.

We recently showed in a mouse model of AAT deficiency how effectively uncontrolled proteolytic activity impaired lung antibacterial immunity in pneumococcal pneumonia, specifically due to NE-mediated degradation of alveolar collectins SP-A and -D [44]. The ability of neutrophil elastase to degrade SP-A and SP-D was also characterized in several other neutrophil dominated diseases [67–71]. Of note, in S100A9 KO mice we also found a strict correlation between release and activity of alveolar NE and degradation of surfactant proteins SP-A and -D. These data again underscore how important a tightly regulated proteolytic response is to inhibit full degradation of lung opsonins, which eventually favors bacterial outgrowth. Based on the currently provided data, we conclude that deficiency of S100A9 (and thus of heterodimeric S100A8/A9) leads to increased concentrations of divalent cations in the pulmonary compartment, which in turn promotes bacterial outgrowth. This at the same time results in increased activation and degranulation of alveolar recruited neutrophils, thereby triggering excess release of neutrophil elastase, which eventually contributes to enzymatic depletion of alveolar collectins SP-A and SP-D, and a subsequent vicious circle with overall fatal consequences for the infected host. (for summary, see Fig 10). Moreover, S100A9 appears to be a promising biomarker to distinguish bacterial from viral pneumonia.

## Material and methods

### Ethics statement

All patients and healthy volunteers enrolled in this study, or their closest relatives provided written informed consent. Collection of biosamples was registered at the German Clinical Trials register (DRKS00000620) and the biosampling was approved by the institutional review board of Hannover Medical School (#2516–2014 and #8146_BO_K_2018).

Handling of animals was done according to institutional guidelines of the Central Animal Facility of Hannover Medical School. All animal experiments were approved by the Lower Saxony State Office for Consumer Protection and Food Safety following the European Council Directive 2010/63/EU and the German Animal Welfare Act.

### Animals

WT (C57BL/6) mice were purchased from Janvier Lab (Le Genest-Saint-Isle, France). S100A9 KO mice on a C57BL/6 background were generated as described elsewhere [32]. Mice used for experiments were matched for age (8–14 weeks) and sex. Experiments were approved by the Lower Saxony State Office for Consumer Protection and Food Safety and were performed according to German laws for animal welfare as well as the EU directive 2010/63/EU.

### Reagents

For pneumococcal growth experiments *in vitro*, MgCl_2_, ZnCl_2_ and CaCl_2_ were purchased from Carl Roth (Karlsruhe, Germany) and MnSO_4_ was purchased from Sigma-Aldrich (Taufkirchen, Germany). For Western blot analysis, polyclonal rabbit anti-mouse SP-A antibody (catalog number ABIN3043205) was purchased from antibodies online (Aachen, Germany). Monoclonal rabbit anti-mouse SP-D antibody (clone ERP 21774–143) was purchased from Abcam (Cambridge, United Kingdom) and monoclonal rat anti-mouse Neutrophil elastase antibody was obtained from R&D Systems (Wiesbaden, Germany). Recombinant S100A8/A9 heterodimer was generously provided by Thomas Vogl, University of Muenster, Germany, or was purchased from BioLegend (San Diego, USA). For immunofluorescence staining, Ly6G AF647 (clone 1A8) and CD11b BV421 (clone M1/70) were purchased from BioLegend (San Diego, USA).

### Study population

Within this study, patients with bacterial and viral ARDS as well as healthy volunteers serving as controls were analyzed. ARDS patients with malignancies and immunosuppression were excluded from the study.

*ARDS patients*: Diagnosis of ARDS was made in accordance with the Berlin 2012 definition [72]. All patients received invasive ventilation at the time of sampling. The study group of patients with bacterial ARDS consisted of 19 patients, including 12 males and 7 females with an average age of 52 (range 22–75 years). Nine of the included patients were smokers and 10 were non-smokers (never or ex-smoker).

The study group of patients with viral ARDS consisted of 11 patients including 8 males and 3 females. The average age was 54 years, with a range from 20–87 years. Six of the included patients were smokers and 5 were non-smokers. All patients underwent BAL for diagnostic reasons.

Seven healthy volunteers were included in this study, all of them were males. The average age was 52 years with a range from 43–59 years. All participants underwent respiratory workup including thorough physical examination, pulmonary function testing and capillary blood gas analysis. All patients were asymptomatic with normal pulmonary function testing and did not receive any medication.

### Bronchoalveolar lavage of ARDS patients and healthy volunteers

BAL was performed in the middle lobe or lingula. BAL samples were rested on ice until timely further laboratory work-up. Macro-impurities in the BAL were removed by filtration of the fluid through sterile gauze. BAL samples were centrifuged at 500 g for 10 minutes at 4°C and subsequently the BAL fluid was aliquoted and stored at -80°C.

### Culture and quantification of *S*. *pneumoniae*

Culture and quantification of *S*. *pneumoniae* was carried out as described [73]. Briefly, aliquots of *S*. *pneumoniae* serotype 19F were inoculated in Todd Hewitt broth (THB, Sigma-Aldrich, St. Louis, USA) supplemented with 20% fetal bovine serum (FCS, Gibco, Thermo Fisher Scientific, Massachusetts, USA) [45,65,74]. Pneumococci were grown to midlog phase, subsequently aliquoted and snap frozen in liquid nitrogen. Until use, bacteria were stored at -80°C. Quantification of *S*. *pneumoniae* was done by plating tenfold serial dilutions onto sheep blood agar plates (BD Biosciences, Heidelberg, Germany) followed by incubation at 37°C and 5% CO_2_ for 18 h. Afterwards colony forming units (CFU) were counted. Additionally, CFU were also determined in mouse inoculation aliquots prior to infection of mice to accurately determine the applied infection dose.

### Protein expression and purification of mutant S100A8/A9 protein

Generation of mutant murine S100 proteins was done analogous to the generation of mutant human S100 proteins, as recently described in detail [43]. S100A8 and S100A9 preferentially form intracellular heterodimers representing the active form of these alarmins. Upon release by activated phagocytes heterodimeric proteins are active in the inflammatory environment but rapidly form inactive S100A8/A9 tetramers. Such tetramerization of S100A8/A9 appears to be an autoinhibitory mechanism. In order to prevent calcium-dependent tetramer formation, we have exchanged an amino acid at distinct sites (N69A and E78A) within the calcium-binding EF-hand II of S100A9, thus creating a mutant murine heterodimeric S100A8/A9 protein. The amino acid exchange does not affect formation of S100A8/A9 heterodimers but prevents calcium-dependent tetramerization, thereby extending its half-life and biological activity. S100 protein expression and purification was done according to established protocols [41–43].

### Infection of mice

Mice were orotracheally infected with *S*. *pneumoniae* (~6 x 10^6^ CFU/mouse) as published elsewhere [73,75]. In brief, mice received a short anesthesia with xylazine (3 mg/kg, Bayer, Leverkusen, Germany) and ketamine (75 mg/kg, CP-Pharma, Burgdorf, Germany). Subsequently, mice were intubated with a 26-gauge Abbocath catheter (Abbott, Wiesbaden, Germany) and the bacterial suspension (in 50 μl PBS) was instilled into the lungs under visual control. Following infection mice were brought back to their cages with free access to food and water. Mice were monitored twice daily for disease symptoms on the basis of an established scoring system and were euthanized according to previously defined criteria.

### Determination of bacterial loads in BAL fluids and lung homogenates

At indicated time points, *S*. *pneumoniae*-infected mice were euthanized and bronchoalveolar lavage (BAL) as well as lung homogenates were prepared as reported earlier [73,76]. Briefly, lungs of mice were rinsed repeatedly with ice-cold phosphate buffered saline (PBS) until a final volume of 1.5 ml plus additional 4.5 ml was reached. Defined aliquots were diluted serially for determination of bacterial loads, while remaining BALF samples were centrifuged for 9 minutes at 1400 rpm and 4°C and BAL cells were harvested for differential leukocyte counts. Lung tissue was homogenized in a total volume of 2 ml Hank’s Balanced Salt Solution (HBSS, Bio&SELL, Nürnberg, Germany) using a tissue homogenizer (IKA, Staufen, Germany). Lung tissue homogenates were filtered (100 μM cell strainer, Greiner, Frickenhausen, Germany) and CFU were determined. Remaining lung tissue homogenates were centrifuged twice for 10 minutes at 13.000 rpm and 4°C and supernatants were stored at -80°C for further analysis.

### Treatment groups

Four treatment groups were established: WT mice were infected with *S*. *pneumoniae* as described above and S100A9 protein levels were determined in plasma and BAL fluid of mice on days 1, 2, 3 and 4 post-infection. The effect of S100A9 deletion on outcome of pneumococcal pneumonia was evaluated in S100A9 KO versus WT mice, which served as disease controls. Mice were infected orotracheally with *S*. *pneumoniae* and bacterial loads as well as lung histopathology were determined at indicated time points. Moreover, disease progression was monitored for an observation period of 6 days.

Four different groups of bone marrow chimeric mice were generated to monitor the effect of hematopoietic deletion or reconstitution of S100A9 on lung CFU and outcome in bone marrow transplanted WT➔WT, KO➔KO, WT➔KO, and KO➔WT mice. Chimeras were infected orotracheally with *S*. *pneumoniae* and bacterial loads were determined at indicated time points. The course of pneumococcal pneumonia was analyzed over a time period of 10 days. In additional experiments, Zn^2+^ levels were determined in lung homogenates of chimeric mice, as indicated.

In selected experiments, the effect of mutant S100A8/A9 protein application on lung bacterial loads in *S*. *pneumoniae*-infected S100A9 KO mice was analyzed. To this end, S100A9 KO mice were orotracheally infected with *S*. *pneumoniae* and treated twice with 50 μg mutant S100A8/A9 protein i.p. or vehicle. Zn^2+^ levels in lungs, lung bacterial clearance and neutrophil recruitment were analyzed at 8 hours after pneumococcal challenge.

### Generation of bone marrow chimeric mice

Generation of bone marrow chimeric mice was done as described earlier [77,78]. Briefly, bone marrow cells were harvested from sex-matched WT and S100A9 KO donor mice under sterile conditions. To this end, tibias and femurs were prepared and flushed with RPMI (Gibco, Thermo Fisher Scientific, Massachusetts USA) supplemented with 10% FCS. Cells were centrifuged for 10 minutes at 1200 rpm at room temperature (RT). Following centrifugation, cell pellets were gently resuspended in Leibovitz L15 Medium (Gibco, Thermo Fisher Scientific, Massachusetts, USA). Cells were filtered through a 40 μm cell strainer (Greiner, Frickenhausen, Germany) to remove cell aggregates and vital cells were counted. For transplantation, recipient WT and S100A9 KO mice received 8 Gy of total body irradiation using a Synergy linear accelerator (Electa, Hamburg, Germany) followed by intravenous injection of 10^7^ bone marrow cells/mouse via lateral tail veins. Recipient chimeric mice were then kept under pathogen-free conditions with free access to sterilized food and water and were monitored twice daily for a time period of 6 weeks. Engraftment efficiencies of more than 97% were accepted for subsequent infection studies [77].

### Analysis of respiratory burst induction in BM-PMN

Analysis of respiratory burst induction in bone-marrow derived neutrophils (BM-PMN) was carried out as described previously [73,79,80]. Briefly, bone-marrow cells were isolated from tibias and femurs of WT and S100A9 KO mice under sterile conditions. Neutrophils were purified using a mouse neutrophil isolation kit (Miltenyi Biotech, Bergisch Gladbach, Germany). Cells (2 x 10^5^) were suspended in 50 μl RPMI/ 10% FCS and seeded into 96-well plates. Following a 30 minute resting time at 37°C/5% CO_2_, 2 mM of luminol was added to each well. Cells were incubated again for 1 hour at 37°C/5% CO_2_ and cells were infected with *S*. *pneumoniae* at a multiplicity of infection (MOI) of 5 to trigger respiratory burst induction followed by monitoring emitted luminescence signals expressed as relative light units (RLU) over time using a Flx800 fluorescence/luminescence reader (BioTec Instruments, Bad Friedrichshall, Germany, KC4 software).

### Phagocytosis assay

Phagocytosis of professional phagocytes from WT and S100A9 KO mice was examined as described recently [79,81]. Shortly, resident alveolar macrophages (rAM) were obtained by bronchoalveolar lavage of untreated WT and S100A9 KO mice. Bone marrow-derived neutrophils (BM-PMN) were isolated as described above. Adherent rAM and highly purified BM-PMN (2 x 10^5^ cells per well) were seeded into 96 well plates and infected with *S*. *pneumoniae* at MOI 26. Following a 30 min incubation in RPMI/10% FCS/1% Glutamine at 37° C and 5% CO_2_, non-phagocytosed bacteria were repeatedly washed off the wells, and residual extracellular bacteria were killed by short incubation of cells in RPMI/10% FCS/1% Glutamine supplemented with 20 μg/ml gentamicin (Sigma-Aldrich, Taufkirchen, Germany). After 10 minutes of incubation at 37° C and 5% CO_2_, cells were washed again and lysed at indicated time points post-infection. Determination of CFU was performed by plating ten-fold serial dilutions of cell lysates onto sheep blood agar plates, followed by incubation for 18 h at 37° C and 5% CO_2_.

### Western Blot analysis

Western Blot analysis was performed as described recently [44,73,76]. Analyses for detection of neutrophil elastase, as well as SP-A and SP-D contents were carried out using cell-free BAL supernatants. Amounts of total proteins per BAL sample were determined by Pierce BCA Protein assay prior to western blot analyses. Accordingly, equal amounts of BAL proteins of untreated and *S*. *pneumoniae-infected* WT and S100A9 KO mice were used to determine the content of SP-A, SP-D and NE in BAL supernatants of mice of the respective treatment groups.

### NE activity assay

Determination of NE activity was done essentially according to recently published protocols [44].

### Casein-based gel zymography

Protein-content of BALF from respective treatment groups was determined using Pierce BCA protein assay kit (Thermo Fisher Scientific, Waltham, USA). Equal protein amounts were supplemented with Laemmli sample buffer, loaded onto casein-containing SDS gels and were run for 1.5 h at 150 V under non-reducing conditions. To get rid of SDS, casein gels were washed twice for 30 min at RT in washing buffer (50 mM Tris-HCl, 5 mM CaCl_2_, 1 μM ZnCl_2_, 2.5% Triton X-100). Next, washing buffer was changed to incubation buffer (50 mM Tris-HCl, 5 mM CaCl_2_, 1 μM ZnCl_2_, 1% Triton X-100) and gels were shaken for 10 min at 37°C. Afterwards, the incubation buffer was changed and gels were shaken again for 18 h at 37°C. Following incubation, casein-containing gels were stained for 30 min with Coomassie Blue G250 (BioRad, Hercules, USA). Subsequently, gels were de-stained until white bands appeared on blue background. White bands within gels therefore represent the location of casein degradation by active BAL fluid proteases.

### Pneumococcal growth experiments

To examine the roles of defined divalent cations on pneumococcal growth *in vitro*, a growth medium depleted of all divalent cations was initially established [22,82–84]. To this end, 5 g/100 ml Chelex 100 Resin (BioRad, Feldkirchen, Germany) was added to THB medium prior to autoclaving. Subsequently, THB-Chelex was stirred overnight at room temperature, followed by sterile filtration over 0.45 μM and 0.20 μM filters (Roth, Karlsruhe, Germany) to remove the resin. THB-Chelex supplemented with 1% FCS served as negative control for all growth experiments, while untreated THB medium supplemented with 1% FCS served as positive control. To examine the impact of different trace metals on pneumococcal growth, magnesium, manganese and zinc were added separately in a dose dependent manner (0 μM– 500 μM) to the medium. THB-Chelex medium (200 μl) supplemented with 1% FCS and the respective trace metals was inoculated with 2 x 10^5^ CFU *S*. *pneumoniae* in triplicates and pneumococcal growth was monitored over a time period of 6 hours. At t = 6 h, samples were serially diluted for determination of bacterial CFU, as described above. In selected experiments, the effect of S100A8/A9 (50 μg/ml) on growth of *S*. *pneumoniae* was analyzed in THB-Chelex medium supplemented with magnesium (200 μM) only, or combinations of magnesium (200 μM) and manganese (50 μM) or magnesium and zinc (50 μM).

### Determination of zinc levels in lung homogenate supernatants

Lung homogenate supernatants were generated as outlined above. Zinc concentrations were analyzed using the LT-SYS Zinc assay (LABOR + TECHNIK, Berlin, Germany) implemented on a Cobas 8000 c502 clinical chemistry analyzer (Roche Diagnostics, Mannheim, Germany).

### Lung histopathology

Lung histopathology was evaluated as described previously [73,76]. Briefly, lungs were filled with 4% formalin fixation medium (Roti-Histofix, Carl Roth, Karlsruhe, Germany) and removed en bloc. After 24 hours of incubation in formalin fixation medium, individual lung lobes were prepared and embedded in paraffin. Lung tissue was cut in 2.5 μm sections, stained with hematoxylin/eosin and analyzed with an Olympus BX53 microscope (Olympus, Tokyo, Japan). Hematoxylin/eosin stained sections of each lung lobe from *S*. *pneumoniae*-infected WT and S100A9 KO mice were examined for the occurrence of interstitial and/or alveolar infiltrating neutrophils and mononuclear cells, hemorrhage as well as interstitial/alveolar edema formation. Percent inflamed lung tissue represents the mean value of lung inflammation recorded for each lung lobe per mouse lung examined.

### Immunofluorescence analysis

Immunofluorescence analysis of frozen lung tissue sections of untreated and *S*. *pneumoniae*-infected WT and S100A9 KO mice was done following a previously published protocol [85]. Briefly, lungs of mice were inflated with 4% paraformaldehyde and 8% saccharose solution and were incubated in saccharose solution overnight. Individual lung lobes were snap frozen in OCT Tissue-Tek (Sakura Finetek, Staufen im Breisgau, Germany). Lung tissue sections (4 μm) were stained with AF647-conjugated anti-Ly6G antibody for identification of neutrophils and with BV421-conjugated anti-CD11b antibody for identification of CD11b expression on Ly6G-positive lung neutrophils. Lung tissue sections were examined using a Zeiss Axio Observer 7 inverted fluorescence microscope equipped with a Zeiss Apotome 2 slider for optical sectioning (Zeiss, Oberkochen, Germany) at x 20 original magnification. Processing of fluorescence images was performed using Zeiss Zen2 Pro software.

### Quantification of macrophages and neutrophils in BAL and lung tissue

Identification and quantification of macrophages and neutrophils recovered from BAL fluids and lung tissue of *S*. *pneumoniae*-infected WT and S100A9 KO mice was done by flow cytometric analysis as described previously [44,81,86]. Briefly, *S*. *pneumoniae*-infected WT and S100A9 KO mice were subjected to BAL, followed by removal of individual lung lobes and digestion of lung tissue with collagenase A (5 mg/ml) and DNAse I (1 mg/ml) for 45 min at 37° C on days 0, 1 and 2 post-infection. For immunophenotypic analysis of macrophages in lung tissue, cell suspensions of CD11c^pos^ cells were enriched using a magnetic cell separation kit (Miltenyi Biotec, Bergisch Gladbach, Germany). Leukocyte subsets purified from BAL fluids and lung tissue were seeded into 96-well plates (5 x 10^5^ cells/well) and treated with Fc receptor blocking agent Kiovig (Baxter AG, Vienna, Austria). Afterwards, cells were stained with fluorochrome-conjugated mAbs specific for F4/80 (anti-F4/80 FITC, clone A3-1, BioRad), Ly6G (anti-Ly6G PE, clone 1A8, BD Biosciences), CD45 (anti-CD45 PE-Cy7, clone 30-F11, BD Biosciences), CD11c (anti-CD11c APC, clone N418, Invitrogen) and CD11b (anti-CD11b BV510, clone M1/70, BD Biosciences) for 20 min at 4°C followed by two washing steps. Subsequently, cells were subjected to FACS analysis of specific cell surface Ag expression using a BD LSR Fortessa flow cytometer (BD Biosciences, San Diego, CA).

### ELISA and Luminex analysis

Human S100A9 and calprotectin levels were measured in plasma and BALF of ARDS patients using commercially available ELISA kits (R&D Systems, Wiesbaden, Germany) according to the manufacturer’s instructions. Mouse S100A9, neutrophil elastase and Surfactant protein D levels were measured in plasma or BALF of untreated and *S*. *pneumoniae*-infected WT and S100A9 KO mice using commercially available ELISA Kits (R&D Systems, Wiesbaden, Germany). Pro- and anti-inflammatory cytokines CXCL1, CXCL2, G-CSF, TNF-α, IL-1β, IL-10 were measured in plasma and BALF of untreated and *S*. *pneumoniae*-infected WT and S100A9 KO mice using Luminex bead arrays (R&D Systems, Wiesbaden, Germany).

### Statistics

Data analysis was performed with GraphPad Prism software and data are shown as mean ± SD. Differences between treatment groups were calculated by Mann-Whitney *U* test or Kruskal-Wallis test. Survival data were analyzed using log-rank test. *P* values lower than 0.05 were assumed significantly different.

## Supporting information

S1 Fig Correlation between BAL fluid S100A9 and C-reactive protein in patients with bacterial or viral pneumonia. (A) Positive correlation between BAL fluid S100A9 and C-reactive protein (CRP) in BAL fluids of patients with bacterial pneumonia (n = 17 patients). (B) Endogenous S100A9 protein in BAL fluids of patients with viral pneumonia was negatively correlated with C-reactive protein (n = 11 patients).
(TIF)

S2 Fig FACS analysis of macrophages and neutrophils in BAL fluid and lung tissue of untreated and *S*. *pneumoniae* infected WT and S100A9 KO mice. WT and S100A9 KO mice were either left untreated or were infected orotracheally with *S*. *pneumoniae*. Macrophages and neutrophils in BALF and lung tissue were analyzed by FACS on days 0, 1 and 2 after pneumococcal challenge. (A,B) Flow cytometric gating strategy for identification of macrophages and neutrophils in BAL fluids of WT and S100A9 KO mice. (A) Macrophages were identified according to their forward scatter area (FSC-A) versus side scatter area (SSC-A) (population 1, P1) and FCS-A/FCS-H characteristics (P2) followed by hierarchical subgating according to their FSC-A versus F4/80 cell surface expression (P3). Alveolar macrophages were characterized as CD11c^pos^/CD11b^neg^ (C) while exudate macrophages were identified as CD11c^pos^/CD11b^pos^ (D). (B) Neutrophils were gated according to their FSC-A/SSC-A profile (P1) followed by hierarchical subgating according to their FSC-A versus Ly6G cell surface expression (P2) and were then identified as Ly6G^pos^/CD11b^pos^ cells (E). (F,G) Flow cytometric gating strategy for identification of macrophages and neutrophils in lungs of WT and S100A9 KO mice. (F) Macrophages were identified according to their FSC-A/SSC-A (P1) and FCS-A/FCS-H characteristics (P2) followed by hierarchical subgating according to their FSC-A versus F4/80 cell surface expression (P3). Lung macrophages were characterized as CD11c^pos^/CD11b^neg^ (H) while exudate macrophages were identified as CD11c^pos^/CD11b^pos^ (I) cells. (G) Neutrophils were gated according to their FSC-A versus SSC-A profile (P1) followed by hierarchical subgating according to their CD45 versus Ly6G cell surface expression (P2) and were then identified as Ly6G^pos^/CD11b^pos^ cells (J). (K) Bacterial loads in BAL fluid of WT and S100A9 KO mice on day 1 and day 2 after pneumococcal challenge. Values are shown as mean ± SD (n = 3–5 mice per time point and treatment group). *p ≤ 0.05, **p ≤ 0.01 compared to WT mice (Mann-Whitney U test).
(TIF)

S3 Fig Analysis of pro- and anti-inflammatory cytokines in BAL fluids of *S*. *pneumoniae* infected WT and S100A9 KO mice. (A-C) Proinflammatory TNF-α (A) and IL-1beta (B) and anti-inflammatory IL-10 (C) cytokine levels in BAL fluids of untreated and *S*. *pneumoniae*-infected WT and S100A9 KO mice on days 1 and 2 post-infection (n = 5–8 mice per time point and treatment group). Data are shown as mean ± SD and are representative of two independently performed experiments. *p ≤ 0.05; **p ≤ 0.01; ***p ≤ 0.001 compared to WT mice (Mann-Whitney *U* test).
(TIF)

S4 Fig Phagocytosis and burst induction in *S*. *pneumoniae*-infected alveolar macrophages and neutrophils of WT and S100A9 KO mice. Cells were purified as described in Materials and Methods followed by infection with *S*. *pneumoniae* at a multiplicity of infection (MOI) of 25. (A,B) Phagocytosis capacity of resident AM (A) and bone marrow-derived neutrophils (BM-PMN) (B) at 30 or 60 minutes after infection of cells with *S*. *pneumoniae*. (C) Burst induction in purified BM-PMN of WT and S100A9 KO mice by *S*. *pneumoniae* (MOI 5). Data are representative of two independently performed experiments.
(TIF)
