# Supplementary figures and images for: S100A9 is indispensable for survival of pneumococcal pneumonia in mice
Source: PLoS Pathog. 2023 Jul 19;19(7):e1011493. doi: 10.1371/journal.ppat.1011493 (PMC10355425; doi:10.1371/journal.ppat.1011493)

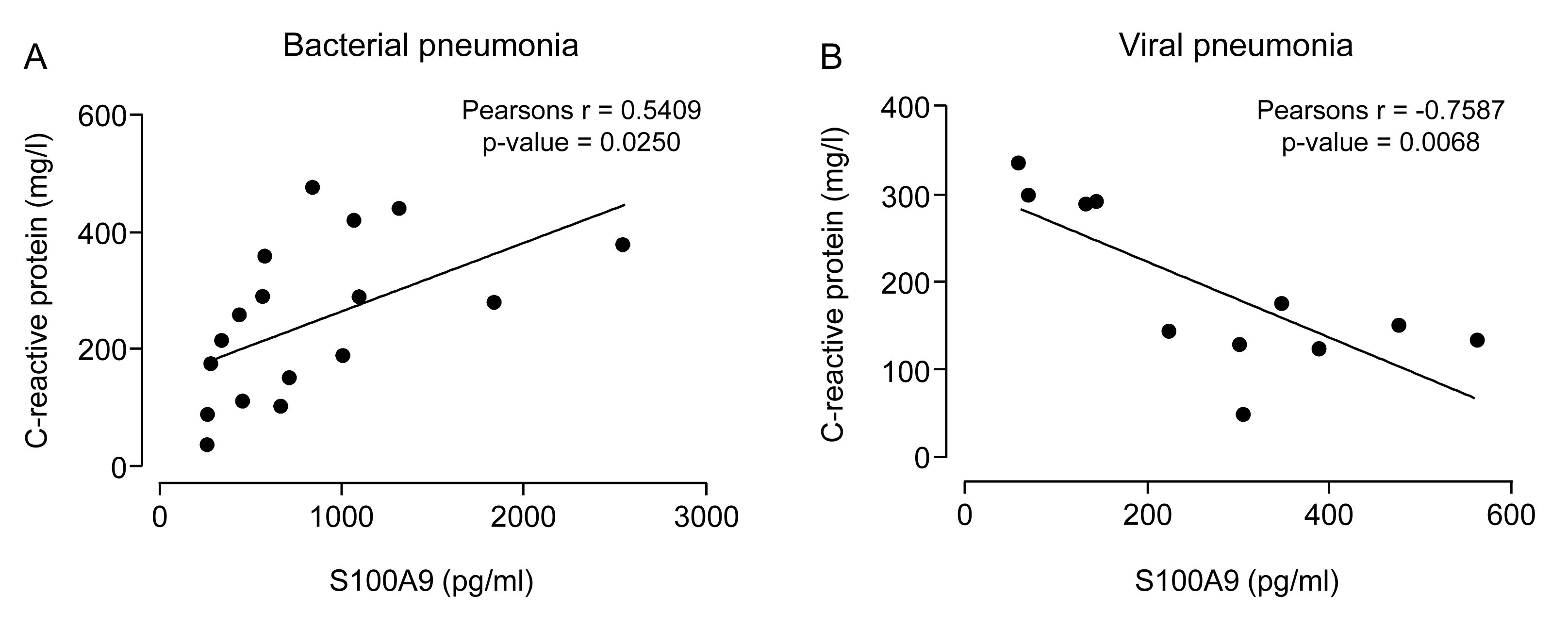

Supplement: S1 Fig — (A) Positive correlation between BAL fluid S100A9 and C-reactive protein (CRP) in BAL fluids of patients with bacterial pneumonia (n = 17 patients). (B) Endogenous S100A9 protein in BAL fluids of patients with viral pneumonia was negatively correlated with C-reactive protein (n = 11 patients). (TIF) [file ppat.1011493.s001.tif]

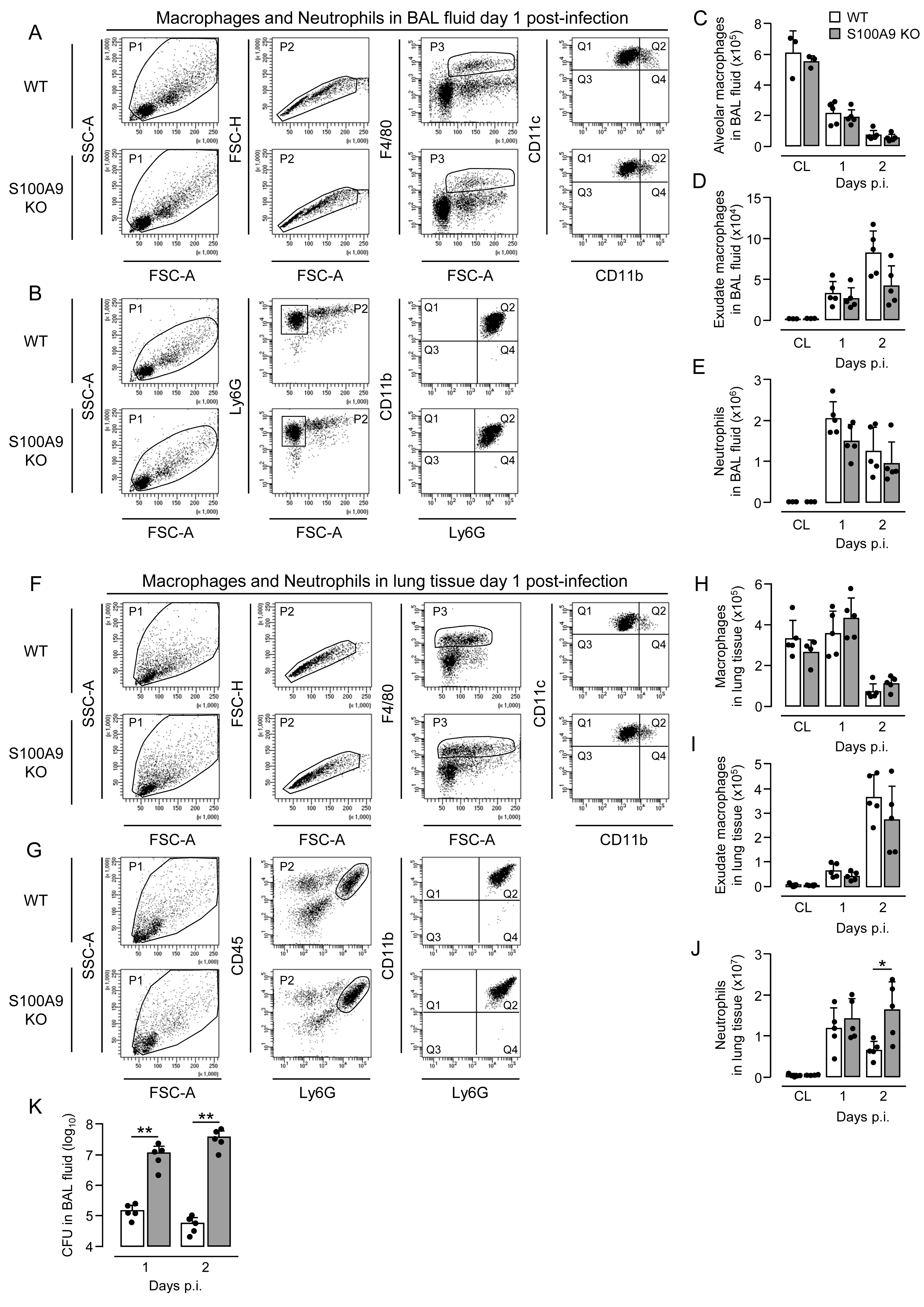

Supplement: S2 Fig — WT and S100A9 KO mice were either left untreated or were infected orotracheally with S. pneumoniae. Macrophages and neutrophils in BALF and lung tissue were analyzed by FACS on days 0, 1 and 2 after pneumococcal challenge. (A,B) Flow cytometric gating strategy for identification of macrophages and neutrophils in BAL fluids of WT and S100A9 KO mice. (A) Macrophages were identified according to their forward scatter area (FSC-A) versus side scatter area (SSC-A) (population 1, P1) and FCS-A/FCS-H characteristics (P2) followed by hierarchical subgating according to their FSC-A versus F4/80 cell surface expression (P3). Alveolar macrophages were characterized as CD11cpos/CD11bneg (C) while exudate macrophages were identified as CD11cpos/CD11bpos (D). (B) Neutrophils were gated according to their FSC-A/SSC-A profile (P1) followed by hierarchical subgating according to their FSC-A versus Ly6G cell surface expression (P2) and were then identified as Ly6Gpos/CD11bpos cells (E). (F,G) Flow cytometric gating strategy for identification of macrophages and neutrophils in lungs of WT and S100A9 KO mice. (F) Macrophages were identified according to their FSC-A/SSC-A (P1) and FCS-A/FCS-H characteristics (P2) followed by hierarchical subgating according to their FSC-A versus F4/80 cell surface expression (P3). Lung macrophages were characterized as CD11cpos/CD11bneg (H) while exudate macrophages were identified as CD11cpos/CD11bpos (I) cells. (G) Neutrophils were gated according to their FSC-A versus SSC-A profile (P1) followed by hierarchical subgating according to their CD45 versus Ly6G cell surface expression (P2) and were then identified as Ly6Gpos/CD11bpos cells (J). (K) Bacterial loads in BAL fluid of WT and S100A9 KO mice on day 1 and day 2 after pneumococcal challenge. Values are shown as mean ± SD (n = 3–5 mice per time point and treatment group). *p ≤ 0.05, **p ≤ 0.01 compared to WT mice (Mann-Whitney U test). (TIF) [file ppat.1011493.s002.tif]

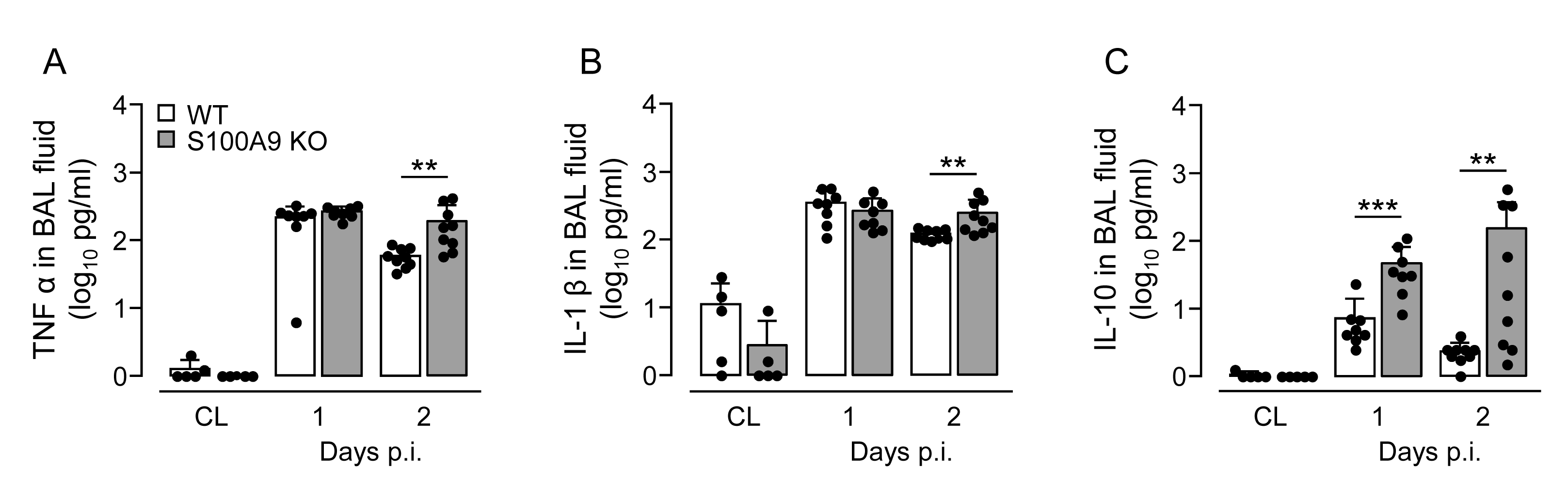

Supplement: S3 Fig — (A-C) Proinflammatory TNF-α (A) and IL-1beta (B) and anti-inflammatory IL-10 (C) cytokine levels in BAL fluids of untreated and S. pneumoniae-infected WT and S100A9 KO mice on days 1 and 2 post-infection (n = 5–8 mice per time point and treatment group). Data are shown as mean ± SD and are representative of two independently performed experiments. *p ≤ 0.05; **p ≤ 0.01; ***p ≤ 0.001 compared to WT mice (Mann-Whitney U test). (TIF) [file ppat.1011493.s003.tif]

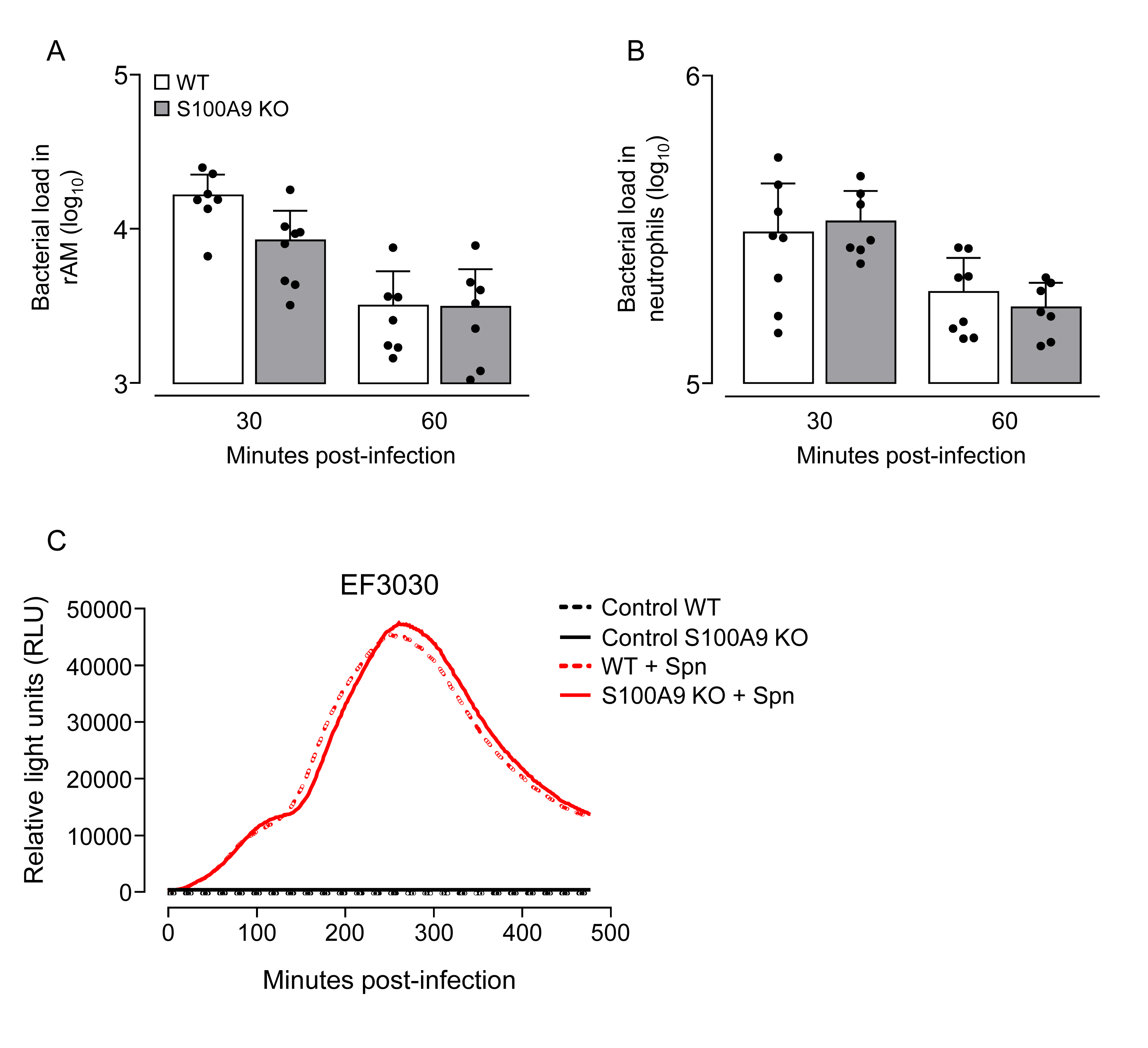

Supplement: S4 Fig — Cells were purified as described in Materials and Methods followed by infection with S. pneumoniae at a multiplicity of infection (MOI) of 25. (A,B) Phagocytosis capacity of resident AM (A) and bone marrow-derived neutrophils (BM-PMN) (B) at 30 or 60 minutes after infection of cells with S. pneumoniae. (C) Burst induction in purified BM-PMN of WT and S100A9 KO mice by S. pneumoniae (MOI 5). Data are representative of two independently performed experiments. (TIF) [file ppat.1011493.s004.tif]
